# British Society of Breast Radiology Annual Scientific Meeting 2016: Manchester, UK. 6-8 November 2016

**DOI:** 10.1186/s13058-016-0766-5

**Published:** 2016-11-08

**Authors:** Ameerah Mohd Azmil, Gaurav Jyoti Bansal, Eleri Davies, Matthew Wallis, Fleur Kilburn-Toppin, Sian Taylor-Phillip, Anne Buckley, Nuala Healy, Aine Quinn, Sylvia O’Keeffe, Teri Ang, Anthony Maxwell, Yit Y. Lim, Elaine Harkness, Richard Emsley, Susan Astley, Soujanya Gadde, Teri Ang, Anthony Maxwell, Yit Y. Lim, Elaine Harkness, Richard Emsley, Susan Astley, Soujanya Gadde, Jennifer Ferguson, Guy Stevens, Robert Hills, Kate Gower Thomas, Sabrina Rajan, Nisha Sharma, Ajit Kishore, John O’ Dowd, Claire Murphy, Ken Lindsay, Guneesh Dadayal, Anne Nielsen Moody, Madeleine Whitaker, Stephen Wolstenhulme, Jane Bull, Nisha Sharma, Anne-Marie Culpan, Natalia Roszkowski, Alexis Grima, Michaela Stahnke, Caroline Rubin, Ruth Walker, Rachel Oeppen, Srikanth Puttagunta, Karen Gray, Shama Puri, Nisha Sharma, Rebecca Millican-Slater, Sana Pascaline, Mohamad Hajaj, Janet Flinn, Kim Thomson, Beatrix Elsberger, Anne Turnbull, Mark Bagnall, Joanne York, Stuart Benney, Amit Goyal, Ashley Topps, Simon Barr, Panagiotis Pikoulas, Susan Pritchard, Anthony Maxwell, Sophie Greenhalgh, Muthyala Sreenivas, Sophie Greenhalgh, Muthyala Sreenivas, Sophie Greenhalgh, Muthyala Sreenivas, Anju Nandhra, Sheetal Sharma, Archita Gulati, Furhan Razzaq, Sana Khan, Jacqueline McKillen, Rupert Larkin, Muthyala Sreenivas, Sachin Kamat, Carla Goncalves, Alan Tan, Asha Eleti, Nithya Vidyaprakash, Michelle A. McMahon, Barbara J. G. Dall, Nisha Sharma, Faisal Majid, Muthyala Sreenivas, Ramachadra Rattehalli, Vandana Gaur, Alistair Mackenzie, Lucy Warren, Mishal Patel, Mark Halling-Brown, Louise Wilkinson, Kenneth Young, James Tanner, Matthew Wallis, Rachael Currie, Sharron Balyes, Julie Wragg, Georgiana Zamfir, Pallavi Ayra, Max Marsden, James Butterworth, Shona Sinha, Anil Desai, Anna Metafa, Madeleine Whitaker, Anne Nielsen-Moody, Stephen Wolstenhulme, Nisha Sharma, Jane Bull, Laura Ward, Samantha Heller, Sue Hudson, Louise Wilkinson, Kathryn Taylor, Argyro Xyda, Anne Turnbull, Steven Allen, Elizabeth Orlowski, Joanne York, Kate Downey, Richard Sidebottom, Matthew Wallis, Ahmad Lodhi, Elaine Harkness, Anthony Maxwell, Anthony Howell, Gareth Evans, Soujanya Gadde, Susan Astley, Yit Lim, Yan Chen, Elizabeth Lepine-Williams, Steven Henderson, Janet Litherland, Naveed Altaf, Yvonne Bury, Nerys Forester, Mei Chan Chin, Janet Litherland, Caroline Taylor, Pippa Skippage, Aneet Sian, Rita McAvinchey, Tom Evans, Guy Stevens, Sian Nisbet, Alex Murray, Kate Gower Thomas, Raquel Clark Castillo, Hannah Markham, Rachel Oeppen, Timothy Thomas, Anil Jain, Jaanilka Molderson, Joseph Wan, Helen Newman, Simon Lowes, Linsley Lunt, Sadia Sultana, Varun Chillal, Muthyala Sreenivas, Rebecca Dalton, David Reeves, Jamie Sergeant, Elaine Harkness, Soujanya Gadde, Anthony Maxwell, Yit Lim, Susan Astley, Liz Edwards, Kate Jenkins, Guy Stevens, Anil Jain, Jaanilka Molderson, Shaheeda Shaikh, Richard Sidebottom, Elizabeth O’Flynn, Laura Bombroffe, Julie Hughes, Erica Scurr, Robin Wilson, Lyn Jones, Sasirekha Govindarajulu, Ajay K. Sahu, Matthew Brown, Steve Allen, Liz O’Flynn, Nandita deSouza, Rumana Rahim, Rema Wasan, Jane Goligher, Shalini Wijesuriya, Juliet Morel, Clare Peacock, Michael Michell, Asif Iqbal, David Evans, Bhavna Batohi, Tavleen Wasan, Vivien Phillips, Eden Manuel, Gillian Bowman, Keshthra Satchithananda, Bhavna Batohi, Cheng Fang, Michael Michell, Juliet Morel, Chirag Shah, Shalini Wijesuriya, Clare Peacock, Rumana Rahim, Rema Wasan, Jane Goligher, Keshthra Satchithananda, Naveed Altaf, Nerys Forester, Leanne Hodkin, Keiran Horgan, David Dodwell, Nisha Sharma, BM Moloney, M. C. Casey, A. Breathnach, R. M. Dwyer, R. McLoughlin, K. J. Sweeney, C. Malone, M. J. Healy, M. J. Kerin, Nerys Forester, Simon Lowes, Elizabeth Mitchell, Maureen Twiddy, Anthony Maxwell, Nisha Sharma, Hilary Dobson, Sarah Vinnicombe, Andrew Evans, David Grant, Briony Bishop, Roxanne Giggens, Shaheel Bhuva, Jennifer Royds, Francesca Camilleri, Gauripriya Babu, Carolyn Beveridge, Rebecca Geach, Katherine Klimczak, Helen Massey, Iain Lyburn, Joseph Wan, Anil Jain, Jaanilka Molderson, Seema Salehi-Bird, Sahithi Nishtala, Liz Gunning, Deepthi Vinayan Changaradil, Jyoti Bansal, Joanna Davis, Ashwini Sharma, Elena Del Voscovo, Harriet Etheridge, Aisling Butler, David S. J. Fenne, Gary Rubin, Helen Burt, Nick Ridley, Sarah Taylor, Muthyala Sreenivas, Subodh Seth, Archana Seth

**Affiliations:** 1 Cardiff University, Cardiff, UK; 2 The Breast Centre, University Hospital of Llandough, Cardiff, UK; 3 Cambridge Breast Unit, Cambridge, UK; 4 University of Warwick, Coventry, UK; 5 Department of Diagnostic Radiology, St. James’s Hospital, Dublin, Ireland; 6 Manchester Medical School, University of Manchester, Manchester, UK; 7 Nightingale Centre and Genesis Prevention Centre, University Hospital of South Manchester, Manchester, UK; 8 Centre for Imaging Sciences, Institute of Population Health, University of Manchester, Manchester, UK; 9 Centre for Biostatistics, Institute of Population Health, University of Manchester, Manchester, UK; 10 Manchester Medical School, University of Manchester, Manchester, UK; 11 Nightingale Centre and Genesis Prevention Centre, University Hospital of South Manchester, Manchester, UK; 12 Centre for Imaging Sciences, Institute of Population Health, University of Manchester, Manchester, UK; 13 Centre for Biostatistics, Institute of Population Health, University of Manchester, Manchester, UK; 14 Breast Test Wales, Cardiff, UK; 15 Cardiff University School of Medicine, Cardiff, UK; 16 Leeds Teaching Hospitals NHS Trust, Leeds, UK; 17 Airedale General Hospital, Keighley/West Yorkshire, UK; 18 Leeds teaching Hospitals NHS Trust, Leeds, UK; 19 University of Leeds, Leeds, UK; 20 University Hospital Southampton, Southampton, UK; 21 Hairmyres Hospital, Lanarkshire, Scotland UK; 22 Royal Derby Hospital, Derby, UK; 23 Leeds Teaching Hospitals, Leeds, UK; 24 Kettering General Hospital, Kettering, UK; 25 Ninewells Hospital, Dundee, UK; 26 University Hospital of South Manchester NHS Foundation Trust, Manchester, UK; 27 University of Manchester, Manchester, UK; 28 University Hospitals of Coventry and Warwickshire, Coventry, West Midlands UK; 29 Warwick Medical School, Coventry, West Midlands UK; 30 Warwick Medical School, Coventry, West Midlands UK; 31 University Hospitals of Coventry and Warwickshire, Coventry, West Midlands UK; 32 Warwick Medical School, Coventry, West Midlands UK; 33 University Hospitals of Coventry and Warwickshire, Coventry, West Midlands UK; 34 Queen Elizabeth Hospital Gateshead, Gateshead, UK; 35 Royal Liverpool University Hospital, Liverpool, UK; 36 North Cheshire Hospitals NHS Trust, Warrington, UK; 37 Ulster Hospital, Belfast, UK; 38 Northern Ireland Medical and Dental Training Agency, Belfast, UK; 39 Warwick Medical School, Coventry, UK; 40 University Hospitals Coventry and Warwickshire NHS Trust, Coventry, UK; 41 Norfolk and Norwich Univerisity Hospital, Norwich, Norfolk UK; 42 Southend University Hospital, Southend Essex, UK; 43 The Leeds Teaching Hospitals NHS Trust, Leeds, UK; 44 UHCW NHS Trust, Coventry, West Midlands UK; 45 Royal Surrey County Hospita, Guildford, UK; 46 St George’s Hospital, London, UK; 47 Cambridge University Hospitals NHS Foundation Trust, Cambridge, UK; 48 University of Surrey, Guildford, UK; 49 InHealth Breast Screening, North and East Devon, UK; 50 Princess Royal University Hospital, Orpington, Kent UK; 51 St James’s University Hospital, Leeds, UK; 52 University of Leeds, Leeds, UK; 53 St George’s Hospital, London, UK; 54 Addenbrookes Hospital, Cambridge, UK; 55 Royal Derby Hospital, Derby, UK; 56 Queen Elizabeth Hospital, Birmingham, UK; 57 Royal Marsden Hospital, London, UK; 58 John Ratcliffe Hospital, Oxford, UK; 59 University of Manchester, Manchester, UK; 60 The Nightingale Centre, University Hospital of South Manchester, Manchester, UK; 61 Loughborough University, Loughborough, UK; 62 Guys and St Thomas NHS Foundation Trust, London, UK; 63 NHS Greater Glasgow & Clyde, Glasgow, UK; 64 West of Scotland Breast Screening Programme, Glasgow, UK; 65 RVI, Newcastle, UK; 66 NHS GGC, Glasgow, UK; 67 Jarvis Breast Screening Centre, Guildford, UK; 68 Breast Test Wales, Cardiff, UK; 69 Royal Gwent Hospital, Newport, UK; 70 All Wales Cancer Genetics Service, Cardiff, UK; 71 University of Southampton, Southampton, UK; 72 University Hospital Southampton, Southampton, UK; 73 The Nightingale Centre and Genesis Prevention Centre, University Hospital of South Manchester, Manchester, UK; 74 The University of Manchester, Manchester, UK; 75 Manchester Academic Health Sciences Centre, Institute of Cancer Sciences, University of Manchester, Manchester, UK; 76 Chelmsford & Colchester Breast Screening Department, Essex, UK; 77 Breast Screening Unit, Queen Elizabeth Hospital, Gateshead, UK; 78 University Hospital Coventry and Warwickshire, Coventry, UK; 79 Manchester Medical School, University of Manchester, Manchester, UK; 80 Centre for Primary Care, University of Manchester, Manchester, UK; 81 Arthritis Research UK Centre for Epidemiology, Centre for Musculoskeletal Research, Manchester Academic Health Science Centre, The University of Manchester, Manchester, UK; 82 NIHR Manchester Musculoskeletal Biomedical Research Unit, Central Manchester University Hospitals NHS Foundation Trust, Manchester, UK; 83 Division of Informatics, Imaging and Data Sciences, School of Health Sciences, University of Manchester, Manchester, UK; 84 Nightingale Centre and Genesis Prevention Centre, University Hospital of South Manchester, Manchester, UK; 85 Breast Test Wales, South East Wales, UK; 86 Public Health Wales, Wales, UK; 87 The Nightingale Centre and Genesis Prevention Centre, University Hospital of South Manchester, Manchester, UK; 88 Manchester Academic Health Sciences Centre, Institute of Cancer Sciences, University of Manchester, Manchester, UK; 89 Royal Marsden NHS Foundation Trust, London, UK; 90 Oxford University Hospitals NHS Foundation Trust, Oxford, UK; 91 Bristol Breast Care Centre, North Bristol NHS Trust, Bristol, UK; 92 The Royal Marsden Hospital, London, UK; 93 The Institute of Cancer Research, London, UK; 94 Kings College Hospital, London, UK; 95 King’s College Hospital, London, UK; 96 RVI, Newcastle, UK; 97 Leeds Teaching Hospitals Trust, Leeds, UK; 98 Department of Surgery, National University of Ireland, Galway, Ireland; 99 Department of Physics, National University of Ireland, Galway, Ireland; 100 RVI, Newcastle, UK; 101 Institute of Health Sciences, Leeds University, Leeds, UK; 102 Nightingale Centre and Genesis Prevention Centre, University Hospital of South Manchester, Manchester, UK; 103 Division of Informatics, Imaging and Data Sciences, School of Health Sciences, University of Manchester, Manchester, UK; 104 Leeds Teaching Hospitals NHS Trust, Leeds, UK; 105 West of Scotland Breast Screening Service, Glasgow, UK; 106 University of Dundee, Ninewells Hospital & Medical School, Dundee, UK; 107 Oxford University Hospitals NHS Foundation Trust, Oxford, UK; 108 Mammography Xray, Edinburgh Breast Unit, Western General Hospital, Edinburgh, UK; 109 Thirlestaine Breast Unit, Cheltenham, UK; 110 University of Manchester, Manchester, UK; 111 The Nightingale Centre and Genesis Prevention Centre, University Hospital of South Manchester, Manchester, UK; 112 Manchester Academic Health Sciences Centre, Institute of Cancer Sciences, Manchester, UK; 113 University Hospital North Midlands, North Midlands, UK; 114 University Hospital Of Llandough, Llandough, South Glamorgan, Wales UK; 115 Western General Hospital, Edinburgh, UK; 116 Neath Port Talbot Hospital, Baglan, UK; 117 The Princess of Wales Hospital, Bridgend, UK; 118 University of Birmingham Medical School, Birmingham, UK; 119 Brighton and Sussex Medical School, Brighton, UK; 120 The Park Centre for Breast Care, Brighton, UK; 121 Breast Unit Great Western Hospital, Swindon, UK; 122 UHCW NHS Trust, Coventry, West Midlands UK; 123 Forth Valley Royal Hospital, Larbert, Stirlingshire UK; 124 West of Scotland Breast Screening Services, Glasgow, UK

## O1 The role of nomograms in predicting axillary node metastasis in breast cancer patients: a comparison between two online predictors and post-operative results

### Ameerah Mohd Azmil^1^, Gaurav Jyoti Bansal^2^, Eleri Davies^2^

#### ^1^Cardiff University, Cardiff, UK; ^2^The Breast Centre, University Hospital of Llandough, Cardiff, UK

##### **Correspondence:** Ameerah Mohd Azmil

**Introduction**

The low sensitivity of ultrasonography (US) in diagnosing axillary lymph node metastasis in breast cancer patients has sparked the evaluation of various tools in an attempt to increase pre-operative sensitivity. We compared axillary lymph node metastasis probability scores with post-surgical findings, using the Memorial Sloan Kettering Cancer Centre (MSKCC) nomogram and Evidencio, two freely available online predictor tools.

**Methods**

We retrospectively evaluated 450 breast cancer patients and analysed data from 194 patients. Ultrasound images were evaluated to assess axillary lymph node status. Patients were divided into groups 0, 1 or ≥2 nodes based on the number of post-operative positive nodes. The difference in mean scores across the 3 nodal groups for both nomograms was analysed using the one-way ANOVA test. The Nottingham Prognostic Index (NPI) was also calculated for each patient. Data was analysed using SPSS ver20 and p<0.05 was considered statistically significant.

**Results**

There were significant differences in mean scores across the 3 nodal groups when using MSKCC (p= 0.000), and Evidencio (p= 0.000). A strong positive correlation was found between MSKCC, Evidencio and NPI. (*r*_*s*_ = 0.671 (MSKCC vs. Evidencio), *r*_*s*_ = 0.721 (Evidencio vs. NPI), *r*_*s*_ = 0.656 (MSKCC vs. NPI), p= 0.000 (for all)).

**Conclusion**

Both MSKCC and Evidencio nomograms can be used to predict axillary node metastasis and guide patient management. Further evaluation is recommended before omitting Sentinel Lymph Node Biopsy or Axillary Node Dissection in patients with very low scores and prompting a ‘second look’ US in patients with high scores.

## O2 Does preoperative axillary staging lead to overtreatment of women with screen detected cancer?

### Matthew Wallis^1^, Fleur Kilburn-Toppin^1^, Sian Taylor-Phillip^2^

#### ^1^Cambridge Breast Unit, Cambridge, UK; ^2^University of Warwick, Coventry, UK

##### **Correspondence:** Matthew Wallis

**Introduction**

Pre-operative staging of the axilla is mandated. In 2011 the ACOSOG Z0011 trial indicated that women with small (T1 – T2) breast cancers and ≤2 nodes positive at SLNB may not require axillary clearance, resulting in a change to surgical practice. Pre-operative ultrasound is still routine despite studies showing between 38% and 46% of symptomatic women with positive pre-operative staging had ≤2 nodes positive at surgery and were thus potentially overtreated or eligible for the POSNOC trial. We measured the impact of current staging in one UK breast screening service.

**Method**

Data were extracted from the unit’s National Breast Screening computer System between 01/04/2008 and 31/03/2015. Axillary staging was compared with final pathology and treatment.

**Results**

164 of 776(21.1%) invasive cancers were node positive. 90 (11.6%) had an axillary biopsy, 54 were positive for cancer (32.9% of the node positive cases). Of these 22 (40.7%) had neoadjuvant treatment, 32 (59.3%) proceeded directly to axillary clearance (mean node count 14.6). 17 (53%) of those who had axillary clearance had ≤2 positive nodes. This compares with 82% of node positive women with a negative biopsy and 74% of node positive women with a normal ultrasound.

**Summary**

This small series suggests significant numbers of women are being denied entry into POSNOC or being potentially overtreated because of routine pre-operative axillary staging. A much larger data set is required to confirm this and predict who would benefit from pre-operative axillary staging. A bid to use the whole ABS data set is with CRUK.

## O3 A comparison of interval breast cancers before and after the introduction of digital screening mammography

### Jennifer Ferguson^2^, Guy Stevens^1^, Robert Hills^2^, Kate Gower Thomas^1^

#### ^1^Breast Test Wales, Cardiff, UK; ^2^Cardiff University School of Medicine, Cardiff, UK

##### **Correspondence:** Jennifer Ferguson

**Introduction**

To determine the rate and classification of interval breast cancer (IC) in the two years preceeding and following the introduction of digital mammography in 2011 in a national screening programme.

**Methods**

Two 2-year bands were used to ensure completed data for pre and post digital groups. Retrospective analysis of a prospectively collected database of IC was undertaken, identifying women who had undergone screening in the two years prior to and after the introduction of digital screening mammography (1.1.10 until 31.12.11 and 1.1.12 until 31.12.13). Chi squared analysis was used to compare the rate between the two groups and the rate of false negative (FN) and minimal signs (MS) categories considered together.

**Results**

Of 87,868 screened before digital between 1.1.10 and 31.12.11, 103 developed IC (0.12%) and of 100,672 screened with digital between 1.1.12 and 31.12.13, 145 developed IC (0.14%) (p = 0.12). Those women with FN and MS interval cancer numbered 17 (16.5%) predigital and 41 (28.3%) with digital; (p = 0.04). The number of true IC was similar in the pre (49.5%) and post (46.9%) groups.

**Conclusion**

The rate if IC is similar to the NHSBSP standard (1.2 per 1000 women screened). Despite earlier groups indicating that digital technology will lessen the number of IC, especially those ‘missed’ (FN and MS groups) which are often asymmetric densities and poorly defined masses, this has not been found in our study. The reasons for this will be discussed and examples will be shown.

## O4 The value of routine screening mammography in women aged 35-39 years in a symptomatic breast unit

### Anne Buckley, Nuala Healy, Aine Quinn, Sylvia O'Keeffe

#### Department of Diagnostic Radiology, St. James's Hospital, Dublin, Ireland

##### **Correspondence:** Anne Buckley

**Introduction**

In the Republic of Ireland, national quality assurance standards state that patients over the age of 35 presenting with breast symptoms should routinely have two-view mammogramography. Our aim was to determine the breast cancer detection rate in women aged 35-39 with low risk factor profiles and normal clinical examinations.

**Methods**

A retrospective analysis of all mammograms performed in patients aged 35-39 at our institution from 2011-2015 was performed. Patients with moderate or high familial risk, previous breast cancer or chest radiation, males, GP and internal hospital referrals, and those with abnormal clinical examinations were excluded. Included women had “normal”, “benign” or undocumented examinations. Results of imaging including subsequent ultrasound and histopathological information was obtained from EPR and PACS systems.

**Results**

4,087 patients aged 35-39 had bilateral mammograms from 2011-2015. 2,149 patients were excluded from analysis.

Of 1,938 included women, 4 (0.21%) were diagnosed with breast cancer confirmed at histology: 2 cases of invasive ductal carcinoma (8 and 2mm) and 2 of DCIS (4.5mm high-grade-DCIS and 2mm low-grade-DCIS). Other histological findings included 2 B3, 40 B2 and 2 B1 lesions resulting from mammographic screening. Overall, 114 biopsies were performed; biopsy rate of 6.09%. 69 (60.53%) were undertaken due to mammographic findings.

**Conclusion**

2.1 cases of cancer were detected per 1000 women screened. This figure would be below accepted international thresholds to undertake screening mammography and raises radiation protection issues. Additionally, a large number of benign biopsies were performed with inherent anticipated psychological impact. Further studies could inform national guidance.

## O5 NHSBSP Prevalent Round Survey: Can we get the recall rate down?

### Sabrina Rajan, Nisha Sharma

#### Leeds Teaching Hospitals NHS Trust, Leeds, UK

##### **Correspondence:** Sabrina Rajan

**Introduction**

NHSBSP standards for assessment recall in prevalent screen is a minimum of <10% with a target of <7%. Aims:

(i) review variation in recall rate
(ii) assess differences in practice and learn from units performing well

**Methods**

Prevalent recall rate for the last 3 years in units were collated and a questionnaire titled ‘NHSBSP Prevalent Round Survey’ designed with units invited to partake on Survey Monkey.

**Results**

Only 24% (19/80) of units achieved a prevalent recall rate of <7% for 3 consecutive years and 25% (20/80) of units had a recall of ≥10% at least once in the last 3 years. In total, 49% (39/80) of units responded:

Reader skill mix and volume

(i) in units with recall of ≥10%, 86% (6/7) have <50% consultant radiologists in film reading team, compared with 22% (7/32) in units with a recall of <10%.
(ii) in 67% (14/21) of units with recall of <7%, all readers achieved target of ≥5,000 mammograms/year, compared with 44% (8/18) in units with ≥7%.

Prevalent arbitration

(i) 5% (2/39) have specific prevalent arbitration with arbitration of all potential recalls.
(ii) 21% (8/39) have a policy for lesion types that do not require recall; 22% (7/32) with recall <10% and 14% (1/7) with recall ≥10%. This includes benign masses, physiological asymmetries and scattered benign calcifications.

**Conclusion**

Units with lower prevalent recall rates have a higher proportion of consultant radiologists and higher volume readers. Policy for lesions that do not require recall can reduce unnecessary assessment of benign lesions.

## O6 Visual assessment of breast density: intra- and inter-observer variability in visual analogue scale scores

### Teri Ang^1^, Anthony Maxwell^2,3^, Yit Y Lim^2^, Elaine Harkness^2,3^, Richard Emsley^4^, Susan Astley^2,3^, Soujanya Gadde^2^

#### ^1^Manchester Medical School, University of Manchester, Manchester, UK; ^2^Nightingale Centre and Genesis Prevention Centre, University Hospital of South Manchester, Manchester, UK; ^3^Centre for Imaging Sciences, Institute of Population Health, University of Manchester, Manchester, UK; ^4^Centre for Biostatistics, Institute of Population Health, University of Manchester, Manchester, UK

##### **Correspondence:** Teri Ang

**Introduction**

Breast density is a strong risk factor for breast cancer and has potential use in breast cancer risk prediction, with subjective methods of density assessment providing a strong relationship with the development of breast cancer. This study aims to assess intra- and inter-observer variability in visual density assessment recorded on Visual Analogue Scales (VAS) among 11 trained readers, and hence the reproducibility over time.

**Methods**

11 readers of varying years of experience estimated the breast density of 120 mammograms on two occasions 3 years apart using VAS. Percent breast density was estimated on VAS score sheets and were scanned using custom software which converted the marks to percentages. Intra- and inter-observer agreement was assessed with intraclass correlation coefficient (ICC) and variation between readers visualised on Bland-Altman plots.

**Results**

Excellent intra-observer agreement (ICC>0.81) was found in majority of the readers. All but one reader had a mean difference <10 percentage points from the first to second reading. Inter-observer agreement was excellent for consistency (ICC 0.82) and substantial for absolute agreement (ICC 0.69). However, the 95% limits of agreement for pairwise differences were (-6.8 to 15.7) at the narrowest and (0.8 to 62.3) at the widest.

**Conclusion**

Overall, the readers were consistent in their scores, although some large inter-observer variations were observed. Reader evaluation and targeted training may alleviate this problem. Intra-observer readings are reliable and may be used in monitoring change in breast density over time, for example when assessing the efficacy of chemopreventive therapies.

## PB.1 Performance and accuracy of pre-operative ultrasound evaluation of axillary lymph nodes in patients with invasive breast cancer

### Ajit Kishore, John O’ Dowd, Claire Murphy, Ken Lindsay, Guneesh Dadayal

#### Airedale General Hospital, Keighley/West Yorkshire, UK

##### **Correspondence:** Guneesh Dadayal

**Introduction**

To assess the performance and accuracy of pre-operative Ultrasound evaluation of axillary lymph nodes in patients with breast cancer.

**Methods**

All patients who underwent wide local excision or mastectomy for invasive primary breast carcinoma in a UK district general hospital between July- Dec 2015 were identified retrospectively and included in the study. Patients who underwent neo-adjuvant chemotherapy were excluded from the study.

**Results**

Out of 94 patients with invasive breast carcinoma included in the study, 38 patients had abnormal axillary nodes on ultrasound and subsequent ultrasound guided axillary nodal biopsy (UANB), of which 16 had metastatic carcinoma from primary invasive breast carcinoma which was later confirmed on axillary nodal clearance (ANC). The remaining 22 patients underwent sentinel lymph node biopsy (SLNB) and had no malignant cells on histology.

Of the 56 patients who had normal pre-operative axillary ultrasound, 11 patients were found to have axillary nodal metastatic disease and 45 patients had no malignant cells on subsequent SLNB.

In total, 27(n=94) patients were found to have axillary nodal metastases in our study.

The positive predictive value of preoperative UANB in detecting axillary nodal metastatic disease is 42% (16/38) and the negative predictive value is 80% (45/56). The sensitivity of axillary ultrasound biopsy is 59% (16/27) and specificity is 67% (45/67). There were 22 false positive cases and 11 false negative cases.

**Conclusion**

The performance and accuracy of pre-operative ultrasound guided axillary nodal biopsy in detecting metastatic axillary nodal disease in our unit remains acceptable within published standards.

## PB.2 Preoperative sentinel lymph node identification, biopsy and localization using contrast enhanced ultrasound (CEUS) in patients with breast cancer, a systematic review and meta-analysis

### Anne Nielsen Moody^1^, Madeleine Whitaker^1^, Stephen Wolstenhulme^1^, Jane Bull^2^, Nisha Sharma^1^, Anne-Marie Culpan^2^

#### ^1^Leeds Teaching Hospitals NHS Trust, Leeds, UK; ^2^University of Leeds, Leeds, UK

##### **Correspondence:** Anne Nielsen Moody

**Introduction**

Sentinel lymph node biopsy (SLNB) is the gold standard for axillary staging. Contrast enhanced ultrasound (CEUS), is a new technique for pre-operative identification, localization and biopsy of SLNs.

**Objective**

Evaluate, in patients with breast cancer and normal axillary B-mode ultrasound, whether: 1) CEUS guided core biopsy of the SLN could identify metastatic nodes pre-operatively and reduce the number of surgical SLNBs. 2) establish whether CEUS SLN identification and localization is a viable alternative to standard lymphatic mapping using isotope and blue dye.

**Methods**

Search of several electronic databases performed. Methodological quality assessed using QUADAS-2. Pooled estimate of sensitivity and specificity for identification of nodal metastases were calculated.

**Results**

Eleven prospective and one retrospective studies with 1520 participants were included. The SLN identification and localization rate for CEUS guided skin marking was 70% - 100%, CEUS guided wire localization 89% - 97% and CEUS guided I-125 seed localization 60%. Across the four studies which evaluated preoperative CEUS guided SLN biopsy, pooled sensitivity for identification of nodal metastases was 54% (95% CI: 47 - 61) and pooled specificity 100% (CI: 99 - 100).

**Conclusion**

CEUS is a promising technique for pre-operative staging of the axilla. CEUS guided core biopsy has the potential to identify nodal metastases in over half (54%) of patients with normal axillary B-mode ultrasound. CEUS guided identification and localization of the SLN may offer a viable alternative to standard lymphatic mapping using isotope and blue dye, however, further prospective studies with larger samples are warranted.

## PB.3 Radiological staging of the axilla: a mixed response?

### Natalia Roszkowski, Alexis Grima, Michaela Stahnke, Caroline Rubin, Ruth Walker, Rachel Oeppen

#### University Hospital Southampton, Southampton, UK

##### **Correspondence**: Natalia Roszkowski

Accurate assessment of axillary nodes in patients with breast cancer influences MDT recommendations about primary therapy, axillary surgery and additional investigations. Ultrasound with image-guided needle biopsy of abnormal nodes is recommended by NICE and cited in the DoH Best Practice Guidelines. Does improved sensitivity of axillary imaging increase the rate of referral for staging investigations?

A completed audit loop evaluated the sensitivity and PPV of axillary ultrasound and image-guided node sampling in consecutive primary breast cancer cases in 2014 and 2012 at the breast imaging unit of a large teaching hospital. Results were compared with published data and the impact on rate of preoperative CT staging and detection of distant metastatic disease was assessed.

Between 2012 (n=47) and 2014 (n=270) overall sensitivity of axillary ultrasound for metastatic nodes increased from 20% to 38%, achieving 95% sensitivity for patients with >1 abnormal node on ultrasound and 61% sensitivity for those with >2 positive nodes at surgery. Between the two audits PPV increased from 40% to 68% and the number of needle biopsies performed significantly increased, yielding a sensitivity of 79% (22/28) in 2014. Referral for pre-treatment staging CT also increased with positivity rate for distant metastases decreasing from 17% to 11% (7/65).

Significantly improved sensitivity and PPV of axillary node ultrasound and needle biopsy are attributed to raised awareness of published data and MDT expectations and feedback. This has resulted in increased referral for pre-operative staging CT with a concomitant decrease in rate of detection of distant metastatic disease.

## PB.4 Axillary scanning at a District General Hospital (DGH) symptomatic breast clinic (SBC) - Audit and review

### Srikanth Puttagunta, Karen Gray

#### Hairmyres Hospital, Lanarkshire, UK

##### **Correspondence:** Srikanth Puttagunta

**Introduction**

Preoperative Ultrasound (US) assessment of axillary nodes is crucial in tailoring management of breast lesions and saves costs and time. US Sensitivity for detection of metastatic axillary lymph nodes ranges from 54.1 % to 68.2%.

At this DGH, cut offs for abnormal axillary lymph nodes were frankly abnormal features or a cortical thickness of 2.5mm or greater.

This audit/review assessed axillary scanning in the context of biopsied suspicious breast lesions at a DGH SBC.

**Methods**

The DGH SBC collects a list of all biopsy cases. Data was obtained using this list, RIS and clinical portal for the time period 1 January 2015- 1 December 2015.

The following ***standard*** was utilised and assessed - “*Ultrasound should identify nodes with metastatic involvement with 50% sensitivity (RCR)*.”

The percentage of breast biopsy cases that also received US axilla assessment at the same time and agreement between axillary US/Biopsy and histopathology were also reviewed.

**Results**

- 179 patients after exclusions with 93 cancer cases.
- Target result: US sensitivity = 63 .2 % (RCR target > 50% sensitivity)
- 96.8% of all malignant cases had their axilla scanned at initial presentation
- 79.5 % Agreement between US and histopathology

**Conclusion**

- This review suggests appropriate scanning of the axilla at this DGH SBC.
- Regular assessment of sensitivity and frequency of axillary scanning still recommended.
- Recommended local improvement of focusing towards performing US in all microcalcification cases- to look for an invasive focus for US biopsy (rather than stereotactic) with axillary scanning at the same time.

## PB.5 Axillary tumour burden in women with early breast cancer and 1 abnormal node on ultrasound scan compared with those with 2 or more abnormal nodes

### Shama Puri^1^, Nisha Sharma^2^, Rebecca Millican-Slater^2^, Sana Pascaline^3^, Mohamad Hajaj^3^, Janet Flinn^4^, Kim Thomson^4^, Beatrix Elsberger^4^, Anne Turnbull^1^, Mark Bagnall^1^, Joanne York^1^, Stuart Benney^1^, Amit Goyal^1^

#### ^1^Royal Derby Hospital, Derby, UK; ^2^Leeds Teaching Hospitals, Leeds, UK; ^3^Kettering General Hospital, Kettering, UK; ^4^Ninewells Hospital, Dundee, UK

##### **Correspondence:** Shama Puri

**Background**

The role of axillary node clearance (ANC) in patients with 1 or 2 positive sentinel nodes is being questioned in the POSNOC trial. Increasing sensitivity of axillary ultrasound has resulted in identifying preoperatively more lymph node positive patients and thereby fewer differences with sentinel node positive patients.

The aim of this study was to determine if the number of abnormal nodes seen on preoperative axillary ultrasound correlates with the axillary tumour burden on histopathology after ANC, and whether this information can be used to identify patients with low volume disease who may be offered sentinel node biopsy(SNB) rather than ANC.

**Methods**

66 patients with FNA or core biopsy proven axillary nodal metastasis were included in this prospective study from 4 centres. The number of abnormal nodes at pre-operative ultrasound examination was recorded (score of ≥3). All patients underwent ANC.

**Results**

31 patients had 1 abnormal node and 35 patients ≥2 abnormal nodes on ultrasound scan. The median number of positive nodes found on pathology was 2(range 1-9) in patients with 1 abnormal node and 5(range 1-33) in patients with ≥2 abnormal nodes (p<.0001). 20 of 31 patients(64.5%) with 1 abnormal node on ultrasound had ≤3 positive nodes at ANC. 18 of 31 patients(58.1%) with 1 abnormal node had only 1 or 2 positive nodes at ANC.

**Conclusion**

Patients with 1 abnormal node seen on ultrasound can be offered SNB rather than ANC as the initial axillary surgery avoiding overtreatment and unnecessary arm morbidity in a significant number of patients.

## PB.6 Preoperative axillary staging in breast cancer: a comparison of the sensitivity of fine needle aspiration biopsy and core needle biopsy

### Ashley Topps^1^, Simon Barr^1^, Panagiotis Pikoulas^1^, Susan Pritchard^1^, Anthony Maxwell^1,2^

#### ^1^University Hospital of South Manchester NHS Foundation Trust, Manchester, UK; ^2^University of Manchester, Manchester, UK

##### **Correspondence:** Ashley Topps

**Introduction**

Identifying axillary lymph node metastases preoperatively can inform discussions about neoadjuvant chemotherapy and also allow a patient to proceed to an axillary lymph node dissection (thus avoiding an additional sentinel node biopsy procedure).

A meta-analysis has not shown a statistically significant difference in sensitivity between axillary US-guided fine needle aspiration biopsy (FNA) and core needle biopsy (CNB)^1^. Moreover, previous studies on this subject have only included low numbers of patients. Our aim was to directly compare the sensitivity of the two techniques.

**Method**

Patients with macrometastic nodal involvement treated at a tertiary referral centre between January 2012 and December 2015 were retrospectively identified from pathology records. The preoperative (first attempt) FNA and CNB results were compared to post-operative histopathology results.

**Results**

A total of 114 CNB's and 229 FNA's were performed. There were 87 true positive CNB's and 147 true positive FNA's. US-guided CNB was therefore more sensitive than US-guided FNA (76.3% vs. 64.2%, *p* = <0.05). There were 9 inadequate results in the CNB group and 16 in the FNA group (7.9% and 7.0%, respectively).A single haematoma requiring non-operative management was recorded in the CNB group.

**Conclusion**

US-guided CNB of the axilla is more sensitive than US-guided FNA and is a safe technique in experienced hands.

**References**

1. Houssami N, Ciatto S, Turner RM, Cody HS, Macaskill P. Preoperative ultrasound-guided needle biopsy of axillary nodes in invasive breast cancer: meta-analysis of its accuracy and utility in staging the axilla. Ann Surg 2011;254(2):243-251.

## PB.7 Potential impact of implementing new ABS guidelines for management of patients with Axillary nodal metastasis on Sentinel Lymph Node biopsy

### Sophie Greenhalgh^2^, Muthyala Sreenivas^1^

#### ^1^University Hospitals of Coventry and Warwickshire, Coventry, UK; ^2^Warwick Medical School, Coventry, UK

##### **Correspondence:** Sophie Greenhalgh

**Background**

In the era of the ACOSOG Z0011 trial, omission of Axillary Lymph Node Clearance (ALNC) in patients with low volume nodal disease and good prognosis cancer is becoming the norm. The Association of Breast Surgeons (ABS) recently produced guidance on axillary management which partially mimic Z0011. The impact of guidance implementation is yet unknown.

**Method**

Patients with newly diagnosed invasive breast cancer, with macrometastasis in 1-2 sentinel nodes plus follow up ALNC, during 2012-2014 at UHCW were selected retrospectively. Inclusion criteria as per ABS guidelines creating 2 patient groups: Good prognosis and bad prognosis cancers. Patients were then grouped into 1-2 or 3 or more nodes based on their total lymph node metastasis burden after ALNC to determine adequacy of proposed treatment. Data was analysed in excel.

**Results**

484 patients underwent Sentinel lymph node biopsy, of which 96 had macrometastasis in 1-2 nodes. 18 patients met the criteria for good prognosis and of these 15 (83.3%) had a total axillary node burden of 1-2 nodes. Only 3 (16.7%) patients had a total of 3 or more positive nodes after ALNC. However, 63 patients met the criteria for bad prognosis, of which only 18 (28.6%) had 3 or more positive nodes after ALNC, the remaining 45 (71.4%) only had a total burden of 1-2 nodes.

**Conclusion**

Implementation of ABS guidelines would have resulted in 16.6% of good prognosis cancers to be undertreated whilst 71.4% of bad prognosis cancers would be over-treated with unnecessary axillary surgery and its resultant morbidity.

## PB.8 Outcomes of patients with Ultrasound detected lymph node metastasis: Are we over-treating the axilla?

### Sophie Greenhalgh^1^, Muthyala Sreenivas^2^

#### ^1^Warwick Medical School, Coventry, UK; ^2^University Hospitals of Coventry and Warwickshire, Coventry, UK

##### **Correspondence:** Sophie Greenhalgh

**Background**

In the era of Z0011, current practice of axillary node clearance for pre-operative US detected lymph node metastasis will result in overtreatment of the axilla as some of these patients only have 1-2 positive nodes.

**Methods**

Patients with lymph node positive, newly diagnosed invasive breast cancer, during 2012-2014 at UHCW were selected retrospectively. Abnormal nodes defined by local criteria underwent ultrasound-guided FNA or core biopsy. Patients were grouped into 1-2 or 3 or more nodes (3+) based on their lymph node metastasis burden after ALNC, and tumour characteristics compared. Categorical variables were evaluated with chi-squared and Fisher’s exact tests.

**Results**

556 patients were diagnosed with invasive cancer, of which 210 had lymph node metastasis. 64 patients were found preoperatively (30.5%) and progressed to ALNC. However, 20 patients (31.3%) had only 1-2 positive nodes. Significantly more in the 1-2 node group underwent breast conserving surgery compared with the 3+ group, 65% versus 25% (p=0.002), and less had T3 cancers, 0% vs 22%, (p=0.024). The 1-2 node group also had a lower proportion of lymphovascular invasion (15% vs. 25%), lobular (10% vs 18%) and grade 3 cancers (20% vs 34.1%) albeit not significant. ER status (80% vs 79.6%) and postmenopausal age (90% vs 88.6%) were similar.

**Conclusion**

Axilla management of patients with pre-operatively diagnosed lymph node metastasis currently leads to overtreatment in 31.3%. There is scope to identify patients with a low nodal burden to select them for axillary staging and possibly avoid ALNC.

## PB.9 The use of nomograms to predict additional lymph node metastasis after Sentinel lymph node biopsy: Can they reliably identify those needing no further axillary treatment?

### Sophie Greenhalgh^1^, Muthyala Sreenivas^2^

#### ^1^Warwick Medical School, Coventry, UK; ^2^University Hospitals of Coventry and Warwickshire, Coventry, UK

##### **Correspondence:** Sophie Greenhalgh

**Background**

Axillary Lymph Node clearance (ALNC) is avoidable in patients with low volume nodal disease. Nomograms from MSK and MD Anderson Cancer Centres (MSKCC and MDA) and Helsinki Hospital are promising predictors of additional node metastasis after Sentinel lymph node biopsy (SNB) but their clinical utility is undetermined.

**Method**

Invasive breast cancer patients newly diagnosed during 2012-2014 at UHCW, with macrometastasis in 1-2 sentinel nodes and ALNC were selected retrospectively. Inclusion criteria: clinically node negative (cN0) and pT1-T2 cancers. Risk scores were calculated using MSKCC, MDA2 and Helsinki nomograms. Patients were grouped based on total nodal burden after ALNC: 1-2 and 3 or more (3+) nodes. Scores between groups were compared with non-parametric independent-sample Mann-Whitney tests. Nomogram utility was evaluated with area under the ROC curve (AUC) analysis.

**Results**

144 patients had a positive SNB, of which 95 patients had cN0, pT1-T2 cancer with macrometastasis in 1-2 nodes. 72 patients (75.8%) had a total 1-2 nodes and 23 patients (24.2%) had 3+ nodes after ALNC. All nomograms were significantly distinguished between patient groups (MDA2 p=0.032, MSKCC p=0.001, Helsinki p=0.030) and AUC gave values and 95% confidence intervals of 0.649 [0.523, 0.775], 0.729 [0.615, 0.843] and 0.651 [0.527, 0.775] for MDA2, MSKCC and Helsinki respectively.

**Conclusion**

Overtreatment with ALNC is 75.8% in patients with sentinel node macrometastasis in 1-2 nodes. MSKCC nomogram had the best utility to identify this group with AUC 0.729. MDA2 and Helsinki nomograms also had good clinical use in deciding which patients should receive axillary clearance.

## PB.10 Is MRI necessary in patients with invasive lobular carcinoma and fatty breasts?

### Anju Nandhra, Sheetal Sharma

#### Queen Elizabeth Hospital Gateshead, Gateshead, UK

##### **Correspondence:** Anju Nandhra

**Introduction**

Invasive lobular cancer (ILC) attributes to 5-10% of new breast cancer diagnosis. It is often mammographically occult with detection rates of 57-87%. Fibroglandular tissue density is inversely correlated to mammographic detection rates and there is also an increased rate of multifocality in ILC. MRI has higher detection rates of 93% and is recommended in patients with ILC. Theoretically mammographic ILC detection should be better in fatty breasts and therefore we ask whether MRI essential in this subgroup of patients?

**Aim**

To ascertain if MRI detects any significant additional lesions in patients with ILC and fatty breasts.

**Methods**

Retrospective study of breast MRIs conducted within a 4 year period for histological confirmed ILC. Breast densities of 1 were included. All imaging reports recorded.

**Results**

134 patients identified with ILC, 103 excluded as had breast density 2 or more. 31 identified as having breast density of 1. Primary lesion identified on mammography in 28 patients and on ultrasound in 3 patients. MRI identified additional findings in 9 patients which were occult on mammography. Of these 9 cases, 3 were identified as additional cancers and the remaining 6 were benign.

**Conclusion**

MRI detected 3 additional cancers which were otherwise occult on mammogram despite the patient having fatty breasts. These results are congruent with published literature. A study published in 2012 identified 7 additional cancers in 32 cases. We therefore conclude that MRI is necessary in imaging patients with ILC and low density breast tissue on mammography.

## PB.11 Is the final post Neoadjuvant chemotherapy MRI scans prior to surgery necessary in patient management in breast cancer?

### Archita Gulati^1,2^, Furhan Razzaq^2^

#### ^1^Royal Liverpool University Hospital, Liverpool, UK; ^2^North Cheshire Hospitals NHS Trust, Warrington, UK

##### **Correspondence:** Archita Gulati

**Introduction**

Neoadjuvant chemotherapy is a well-established method of treating large breast carcinomas with the aim of shrinking the tumour to enable breast conservation surgery or to improve outcomes of treatment for patients requiring mastectomy by delivering early systemic therapy to reduce the risk of metastatic disease.

In many institutions, MRI scans are performed prior to the commencement of neoadjuvant chemotherapy, at the mid-point of treatment and at the end of chemotherapy prior to surgery. The aim of this audit was to assess the benefit of performing the final MRI scan in the patient's surgical management.

**Methods**

A list of relevant patients undergoing neoadjuvant chemotherapy was obtained from a database maintained by the breast care nurses. Tumour size on the final MRI scan was compared with tumour size determined histologically from the operative specimen, which was regarded as the gold standard for the purposes of this audit. Concordance was regarded as satisfactory if the tumour size was within 10mm on radiology and histology.

**Results**

There was 77% concordance between the final MRI scan and the postoperative histology. However the findings at MRI did not alter the surgical management decision and in patients where there was discordance in tumour size between imaging and histology, none required further surgery.

**Conclusions**

Although felt to be useful by some surgeons and oncologists, MRI scans post neoadjuvant chemotherapy prior to surgery can be safely omitted without adversely affecting the patient's management.

## PB.13 Additional significant findings in pre-operative breast MRI in patients with histologically proven breast carcinoma- our experience in one year

### Sana Khan^1,2^, Jacqueline McKillen^1^

#### ^1^Ulster Hospital, Belfast, UK; ^2^Northern Ireland Medical and Dental Training Agency, Belfast, UK

##### **Correspondence:** Sana Khan

**Aim**

To evaluate additional findings on patients undergoing breast MRI for pre-operative planning.

**Methods**

Our unit conducted 73 breast MRI studies between 30/06/2015 and 30/06/2016. These were performed for various indications; multifocality, disease extent in lobular carcinomas, mammographically occult cancers, young patients with breast cancer and dense breast tissue. Age ranged from 31-70 years. Histology was correlated for all positive findings.

**Results**

Total of 27% scans had relevant findings which required further investigations. Benign lesions contributed 20% to the relevant findings, 5% of these required change in management.

Sinister findings were depicted in 80% of all the relevant findings. Metastases contributed 30% to the relevant findings. In terms of disease extent or localisation identified with high clinical suspicion and negative imaging, 20% had mammographically occult carcinomas, multifocal disease was present in 20%, 1/20 had diffuse disease and 1/20 was positive for contralateral carcinoma.

In summary, management was changed in 23.2% of all the scans.

**Conclusion**

Breast MRI does demonstrate significant additional lesions. Though these warrant further imaging, patient management and MDM recommendation may alter which is proven in 23.2% of cases in our study.

## PB.14 Monitoring response in breast cancer patients undergoing neoadjuvant chemotherapy - a single institution experience

### Rupert Larkin^1^, Muthyala Sreenivas^2^

#### ^1^Warwick Medical School, Coventry, UK; ^2^University Hospitals Coventry and Warwickshire NHS Trust, Coventry, UK

##### **Correspondence:** Rupert Larkin

**Background**

Dynamic Contrast Enhanced Magnetic Resonance Imaging (DCE-MRI) is used to monitor tumour response to neoadjuvant chemotherapy (NAC) for locally advanced breast cancer (LABC) through analysis of tumour morphology (size and shape) and contrast kinetic patterns. This study aimed to identify pre-NAC MRI and tumour features which could help predict response to NAC. We also investigated the ability of post-NAC MRI to accurately represent the extent of any residual disease which is of importance for surgical planning.

**Methods**

This retrospective cohort study evaluated forty-six patients with LABC who received NAC between 2008 and 2016 at UHCW. All patients underwent pre and post-NAC DCE-MRI and proceeded to surgical excision. Clinical and imaging data was collected from CRRS and UHCW PACS.

**Results**

Several tumour features were found to be predictive of pathological response, including: tumour morphology, oedema, shrinkage pattern, HR status, HER2 status and percentage change in MRI long diameter. Circumscribed lesions were more likely respond to NAC than irregular, diffuse and nodular tumours. There was some evidence of correlation between MRI morphological categories and tumour receptor status. The accuracy of post-NAC MRI for predicting residual disease was superior for HR negative tumours.

**Conclusions**

Pre-treatment MRI features can serve as reliable imaging biomarkers which can be used to predict disease response to NAC. The accuracy of post-NAC MRI varies with tumour biology and imaging features, and this should be considered in decisions about surgical approach.

## PB.15 Comparison of digital mammography, ultrasound and MRI in the preoperative size assessment of lobular breast cancer: A district general hospital experience

### Sachin Kamat^1^, Carla Goncalves^2^, Alan Tan^2^, Asha Eleti^2^, Nithya Vidyaprakash^2^

#### ^1^Norfolk and Norwich Univerisity Hospital, Norwich, UK; ^2^Southend University Hospital, Southend, UK

##### **Correspondence:** Sachin Kamat

**Introduction**

Tumour size assessment with imaging has an impact on the diagnosis and treatment planning of breast malignancy. We assessed the correlation between preoperative size of lobular breast cancer on different imaging modalities and postoperative histological size, as the reference standard.

**Methods**

Retrospective review of lesion size on digital mammography (DM), ultrasound (US) and contrast-enhanced MRI from 73 breast lesions was performed in 63 consecutive women with histological diagnosis of lobular breast cancer. The sizes of different modalities were correlated with histological tumour size using paired T-test analysis and coefficient of determination.

**Results**

Of the 63 women included, 3 (5%) had bilateral lesions and 7 (11%) had 2 foci in the same breast; 73 separate lesions were entered in the analysis.

The mean tumour sizes were: histological = 21 mm (SD = +/- 16mm), DM = 19 mm (SD = +/-15mm), US = 14mm (SD = +/- 12mm), MRI = 20 mm (SD = +/- 16mm).

The means of imaging modality minus the histology size were: MRI -0.85 (95%CI: -2.87 to 1.17, p-value= 0.4042), DM -1.77 (95%CI: -4.43 to 0.89, p-value= 0.1895) and US -6.77 (95%CI: -9.06 to -4.47, p-value= 0. 0.0001).

When compared to histology, coefficients of determination (R-squared) were: MRI 0.72, US 0.62 and DM 0.53.

**Conclusion**

There is no statistically significant size difference between MRI and histological size. Furthermore, correlation between the two is high. In comparison with DR and US, MRI is a reliable imaging modality in preoperative assessment of lobular breast cancer size.

## PB.16 Pragmatic use of breast MRI in pre-operative planning

### Michelle A McMahon, Barbara JG Dall, Nisha Sharma

#### The Leeds Teaching Hospitals NHS Trust, Leeds, UK

##### **Correspondence:** Barbara JG Dall

**Introduction**

MRI is under scrutiny and the new NICE guidance states that all breast cancers should not have an MRI unless clear indication. We reviewed our practice as an experienced unit.

**Methods**

All cases where MRI performed to plan surgery were identified in the calendar year 2014. Type and number of operations and whether 2nd look ultrasound performed noted.

**Results**

Approximately 600 breast cancers per year (symptomatic/screening).

103 Breast MRI's (17% total cancers).

13 cases excluded as no surgery/NACT/incomplete information.

Total 90 cases, 35 for extent of lobular, 46 other reasons - size mismatch/? MF/MC/mammo occult/dense mammogram and 9 for high grade DCIS.

72/90(80%) went straight to surgery - 51(71%) WLE, (45 single operation) and 21(29%) Mastectomy.

18/90(20%) had a 2nd look Ultrasound and 14/18 had biopsies(10 US, 3 MRI, 1 Diagnostic). 9/14 malignant and 5/14 benign.

Of the 9 malignant biopsies, 8 in the ipsilateral and 1contralateral breast.

Of those who went to Mastectomy 7 had 2nd look Ultrasound and biopsy (1 contralateral breast) and 2 had 2nd look ultrasound and no biopsy.

**Conclusion**

17% of our cases had MRI showing that we are adhering to the NICE standard. In 60% of cases MRI allowed for successful single conservative operation. MRI allows picturisation of tumour morphology e.g. 2 discrete lesions or 2 lesions with extensive disease between them, aiding appropriate surgery. MRI needs to be reported by expert breast radiologists and interpreted within the clinical context by the MDT team.

## PB.17 Feasibility of utilising FAST protocol for clients undergoing NHSBSP high risk MRI scans

### Faisal Majid, Muthyala Sreenivas, Ramachadra Rattehalli, Vandana Gaur

#### UHCW NHS Trust, Coventry, UK

##### **Correspondence:** Faisal Majid

**Aim**

Both performing and reporting breast MRI scans is time consuming and expensive. An alternate method for imaging and reporting which achieved excellent results utilising a shortened protocol and rapid reporting was reported by C. Khul et al. This retrospective study was used to test the feasibility of such protocol in a centre which is starting to perform high risk breast MRI screening.

**Methods**

Three consultant radiologists (C1, C2 and C3 with 7 to 9 years of experience in reading breast MRI) retrospectively and independently reviewed 27 high risk breast MRI studies. All cases were annonymised and review was only performed as per C. Khul's paper, that is MIP images and abbreviated protocol images consisting of pre-contrast, first post-contrast and its subtracted counterpart were only reviewed and reported. Time taken for MIP and abbreviated protocol reading and the sensitivity and specificity for both were calculated for each reader.

**Results**

Average time to read MIP and abbreviated protocol images were: C1 = 1.8 and 26.3 sec, C2 = 2.6 and 8.1 sec and C3 = 5.3 and 63.7 sec. Only one patient had cancer (bilateral cancer) and imaging finding were scored R3 and R4 for right and R5 for left by all readers. Therefore, no cancer was missed. Altogether 7 cases underwent 2^nd^ look US and found to have benign results.

**Conclusion**

This small study shows that the method reported by C. Khul is feasible and should result in lower cost service. Prospective larger scale UK study should be considered.

## PB.20 Characterisation of cancers from digital mammograms from a large scale database: size, conspicuity and appearance

### Alistair Mackenzie^1^, Lucy Warren^1^, Mishal Patel^1^, Mark Halling-Brown^1^, Louise Wilkinson^2^, Kenneth Young^1,4^, James Tanner^3^, Matthew Wallis^3^

#### ^1^Royal Surrey County Hospital, Guildford, UK; ^2^St George's Hospital, London, UK; ^3^Cambridge University Hospitals NHS Foundation Trust, Cambridge, UK; ^4^University of Surrey, Guildford, UK

##### **Correspondence:** Alistair Mackenzie

**Introduction**

There is a lack of collated data on the appearance of cancers in digital mammograms. This information may help in understanding which lesions appearing in mammograms are potentially fatal and which low risk.

**Methods**

Mammograms have been collected since March 2011 from three breast screening sites alongside the clinical information from National Breast Screening Service (NBSS). Experienced readers have marked a rectangular region of interest (ROI) closely around identified cancers and classified each cancer's conspicuity and appearance. The cancers size was calculated as maximum dimension (height or width) of the ROI. The data were analysed to investigate the appearance of the cancers.

**Results**

Currently, there are 4657 women's images in the database with invasive and in-situ cancers. 3400 of the cancers have been marked with complete data. Most of the cancers were described with an obvious conspicuity (67%) rather than subtle or very subtle. It was found that 10%, 22%, 21%, and 47% of the cancers were in the following mammographic size ranges ≤10mm, 10-15mm, 15-20mm, >20mm respectively. The most common cancer appearance was masses (50%), followed by calcification clusters (28%), asymmetric densities (13%) and architectural distortions (9%). The masses were classified as ill-defined (58%), spiculated (38%) and well defined (4%), also 14% of them had associated calcifications.

**Conclusion**

We have characterised the appearance of a large set of cancers. Once the database is fully linked to data in NBSS, then cancer grade and biology can be included in the analysis.

## PB.21 Improvement in cancer detection rates following replacement of older digital mammography equipment with new digital

### Rachael Currie, Sharron Balyes, Julie Wragg, Georgiana Zamfir

#### InHealth Breast Screening, North and East Devon, UK

##### **Correspondence:** Rachael Currie

**Aim**

There is evidence that digital mammography improves breast cancer and non-invasive detection rates when compared to analogue. This has been the case as breast units across the country have changed over to digital equipment. Does replacement of older digital for new digital equipment further increase cancer detection rates?

**Method**

The North and East Devon Breast Screening unit run by InHealth was the first unit in the country to become fully digital in2006. Thus we will have been the first unit to replace all our digital equipment for new, carried out in 2013. We have reviewed cancer rates for the final year with old digital equipment and the 2 years post replacement.

**Results**

We have shown an increase in cancer detection rates following replacement of digital equipment. Non-invasive detection rate per 1000 screened has increased from 1.68 to 2.55 and 2.25. Small invasive cancer rate has increased from 2.66 to 2.81 and 3.12. Tables document these results for invasive, non-invasive and <15mm cancers for prevalent and incident groups over 3 years.

**Conclusion**

Replacement of digital mammography equipment for new increases cancer detection rates particularly in non-invasive and small cancers.

## PB.22 Does the mammographic spicule size improve correlation with histological size in invasive breast cancer?

### Pallavi Ayra, Max Marsden, James Butterworth, Shona Sinha, Anil Desai, Anna Metafa

#### Princess Royal University Hospital, Orpington, UK

##### **Correspondence:** Pallavi Ayra

**Introduction**

Mammography has been variously reported as both overestimating and overestimating the size of breast cancer when compared to histology. This study aimed to determine whether the inclusion of spicules when measuring the cancer reduces the discordance between the two methods.

**Methods**

Data from 91 patients with either invasive ductal carcinoma (IDC) or invasive lobular carcinoma (ILC) were analysed in a retrospective study from January 2014 to March 2016. Histological tumour size was assessed with and without ductal carcinoma in situ (DCIS) for the overall tumour size and similarly mammographic size was measured with and without spicules. The largest tumour diameter was chosen in each case. Correlation was assessed using Spearman's rho. A Bland-Altman analysis was used to assess the agreement between the mammographic and histological sizing techniques.

**Results**

76 patients had IDC and 15 patients had ILC. The mean difference between IDC + DCIS histology and mammographic with spicule was -5.7mm (+/-10.6mm), r = 0.72. The mean difference between IDC + DCIS histology and mammographic without spicule was 4.2mm (+/-10.9mm), r = 0.68. The mean difference between ILC histology and mammographic with spicule was -7.9mm (+/- 10.0mm), r = 0.73. The mean difference between ILC histology and mammographic without spicule was 3.1mm (+/-5.7mm), r = 0.82. A Bland-Altman analysis revealed only ILC without DCIS histology and mammography without including spicules had good agreement.

**Conclusion**

The inclusion of mammographic spicules over estimates the final histology size for both IDC and ILC without improving the correlation between the two methods.

## PB.23 Systematic review comparing breast cancer detection of contrast enhanced spectral mammography with full field digital mammography

### Madeleine Whitaker^1^, Anne Nielsen-Moody^1^, Stephen Wolstenhulme^1^, Nisha Sharma^1^, Jane Bull^2^

#### ^1^St James's University Hospital, Leeds, UK; ^2^University of Leeds, Leeds, UK

##### **Correspondence:** Madeleine Whitaker

**Introduction**

Full field digital mammography (FFDM) is currently the gold-standard breast imaging modality for suspected breast cancer, however it has limitations imaging dense breasts. Contrast enhanced spectral mammography (CESM) is a new technique which utilises dual energy exposures with iodinated contrast media to highlight areas of angiogenesis, a characteristic of malignancy, on mammography images. This could improve the diagnostic accuracy of mammography.

**Objectives**

Undertake a systematic review comparing CESM with FFDM, to establish whether sensitivity and specificity of breast cancer detection is significantly improved.

**Methods**

A comprehensive search of journal databases and grey literature sources was performed using multiple terms for CESM, FFDM and breast cancer to identify relevant literature. These studies were screened against pre-determined inclusion and exclusion criteria to ensure they were applicable to the review question. The included studies were quality assessed and their data extracted.

**Results**

Eleven relevant studies were identified. These performed the same CESM technology (G.E. SenoBright®) and varied mammography technologies on the same patients with either newly diagnosed breast cancer or suspicious imaging. All studies showed CESM increased sensitivity by 3.1-21.2% and 10 studies showed increased specificity by 5-45.7% (one study found specificity decreased by 4%) compared to mammography. However the differences were not always significant, the data was heterogeneous, and none of the included studies fully demonstrated low risk of bias when quality assessed.

**Conclusion**

CESM may improve sensitivity and specificity of breast cancer detection compared to mammography. However further research is required to establish applications and determine which patients would benefit.

## PB.24 Effect of parenchymal pattern in women with dense breasts, variation with age and impact on screening outcomes - observations from a UK screening programme

### Laura Ward, Samantha Heller, Sue Hudson, Louise Wilkinson

#### St George's Hospital, London, UK

##### **Correspondence:** Louise Wilkinson

**Introduction**

To analyse the pattern of parenchymal tissue on mammography in women with the densest breasts, variation with age and the effect on recall rates and cancer detection.

**Methods**

Breast density data (VolparaTM) was obtained in women attending mammographic screening between April 2013 and March 2015. Cases with the densest breasts were selected for visual interpretation of parenchymal pattern. 100 cases were included for non-assessed women age 50, 55, 60, 65 and 69-71. All cases of assessed women with the densest breasts were reviewed. Mammograms were reviewed by 10 film readers. Parenchymal pattern was classified as: smooth; mainly smooth, mixed; mainly nodular or nodular.

Average classification was compared by age and assessed v non-assessed. Likelihood of biopsy and cancer diagnosis was analysed by parenchymal pattern.

**Results**

40760 women were included in the subset, 4331 (10.6%) of these were Volpara4. Proportions in each parenchymal pattern category were similar at all ages and for assessed v non-assessed.

In the assessed group 90 were smooth/mainly smooth; 104 mixed; 106 mainly nodular/nodular. Of women who were subjected to biopsy, 50 were smooth/mainly smooth; 57 mixed; 56 mainly nodular/nodular. Of women diagnosed with cancer, 7 were smooth/mainly smooth; 10 mixed; 18 mainly nodular/nodular. More cancers were identified in women with nodular breasts.

**Conclusion**

The ratio of smooth to nodular pattern in women with the densest breasts did not vary with age. The parenchymal pattern of breast tissue did not affect recall rate, but women with nodular breasts were more likely to be diagnosed with cancer.

## PB.25 Presentation and follow up of mammographically occult breast cancer: a multicentre audit of 5 years presentation with minimum 5 years follow up

### Kathryn Taylor^1^, Argyro Xyda^3^, Anne Turnbull^2^, Steven Allen^4^, Elizabeth Orlowski^1^, Joanne York^2^, Kate Downey^4^, Richard Sidebottom^5^, Matthew Wallis^1^

#### ^1^Addenbrookes Hospital, Cambridge, UK; ^2^Royal Derby Hospital, Derby, UK; ^3^Queen Elizabeth Hospital, Birmingham, UK; ^4^Royal Marsden Hospital, London, UK; ^5^John Ratcliffe Hospital, Oxford, UK

##### **Correspondence:** Kathryn Taylor

**Introduction**

Approximately 10% of symptomatic female breast cancers (BC) are mammographically occult (MO) yet data is scarce regarding presentation and histology of the primary and any recurrence. It is unknown whether mammographic follow-up is appropriate. We are auditing women diagnosed with symptomatic MOBC across 3 UK centres, to increase the knowledge base and inform future surveillance methods. We present preliminary results.

**Method**

For each centre women ≥ 35 attending as GP referred between 2006 and 2010, undergoing mammograms and diagnosed with a symptomatic MOBC were retrospectively identified. Presentation, histology and treatment data was collected. Type/frequency of imaging follow-up and subsequent recurrences to 31.12.15 were documented. Each MOBC identified was age matched with 2 women with a mammography visible cancer.

**Results**

116 MOBC were identified across 2 centres (332 controls), average age 53yrs. 76.5% presented with a lump vs 81.6% in the control group. 37.3% vs 17.5% had very dense parenchyma. Mean size at diagnosis was 15.5mm (range 4-37) vs 24.0mm (range 4-90). 11.1% vs 9% were lobular. 62.6% vs 51% were node negative 91.1% vs 90.3% were ER+ve and 11.2% vs 20% were HER 2+ve. 52.4% vs 33.4% underwent breast conservation. Mammographic follow up was similar for each group; median 5.4yrs (range 0.4-9.9). All 6 recurrences to date were visible on mammography.

**Conclusion**

Preliminary data suggests that MOSCs are more common in dense breasts, but smaller at diagnosis favouring surgical conservation. Recurrences are not necessarily occult so mammographic follow-up may be appropriate. Further data is being collated to consolidate findings.

## PB.26 Mammographic density changes over two screening rounds: Effect of BMI, menopausal status and HRT use

### Ahmad Lodhi^1^, Elaine Harkness^1,2^, Anthony Maxwell^1,2^, Anthony Howell^1,2^, Gareth Evans^1,2^, Soujanya Gadde^2^, Susan Astley^1,2^, Yit Lim^1,2^

#### ^1^University of Manchester, Manchester, UK; ^2^The Nightingale Centre, University Hospital of South Manchester, Manchester, UK

##### **Correspondence:** Ahmad Lodhi

**Introduction**

Mammographic density (MD) is the strongest modifiable risk factor for breast cancer. We studied MD changes over a 3-year period and the effect BMI, menopausal status and HRT had.

**Methods**

Recruitment included 5751 women who participated in the Predicting Risk of Cancer At Screening (PROCAS) study and had a second routine screen. BMI, menopausal status and HRT use were self-reported at first PROCAS screen. Fibroglandular volume (FGV, an absolute measure for MD) was measured using an automated volumetric method (Volpara^TM^) at both screens.

**Results**

Average starting FGV was 49.951 cm^3^ and decreased 0.474 cm^3^ over the 3-year period. However, 48.7% of women demonstrated an increase in FGV. Women with normal BMI gained on average 0.557 cm^3^ in FGV. As BMI increased the average FGV decreased with obese women losing on average 2.256 cm^3^. Women who were pre/perimenopausal at first screening had larger drops in FGV than postmenopausal women. Within postmenopausal women 49.4% saw rises in FGV and of those who did the average gain was 8.562 cm^3^. The use of HRT showed an expected positive correlation with FGV.

**Conclusion**

There was an overall decrease in MD over the 3-year period. However, 48.7% of women demonstrated an increase in MD; in particular women with normal BMI, postmenopausal women and women on HRT. These data show that to fully understand changes in individual MD for both breast cancer risk prediction and risk reducing interventions, confounding factors must be taken into account.

## PB.27 How new readers perform as compared to more experienced readers on the PERFORMS scheme

### Yan Chen

#### Loughborough University, Loughborough, UK

**Introduction**

Breast screening in the UK is undergoing many workforce changes as more experienced radiologists retire and new readers become engaged. With such major changes it is important to monitor mammographic interpretation skills for national quality assurance purposes. A detailed analysis has been carried out to compare the performance of ‘newcomers’ to the national performance measures on the PERFORMS scheme of more experienced readers.

**Methods**

All UK screening readers take part annually in the PERFORMs educational scheme. 59 new participants read a recent set of PERFORMS cases. Nationally, 726 more experienced readers also read the difficult same cases.

**Results**

A two-tailed t-test indicated that the mean of cancer detection of new participants was significantly lower as compared to the national average of more experienced participants (p<0.0001, M_new_=72.35% M_national_=85.82%). The correct recall (sensitivity) of the new participants was also significantly lower than the national average (p<.000001, M_new_=72.88%, M_national_=85.92%). Additionally, the correct return to screening (specificity) of the new participants was significantly lower than the national average (p<.000001, M_new_=85.86%, M_national_=83.55%).

**Conclusions**

On several performance measures, new breast screening readers performed significantly worse than more experienced readers. This may partly be due to them being new to taking part in the PERFORMS scheme but that cannot fully explain the very significant differences found. The purpose for the PERFORMS scheme is to highlight such performance differences and to then help new, and poor performers, improve and maintain their performance so that the quality of the National Screening Programme remains high despite workforce changes.

## PB.28 Contrast Enhanced Spectral Mammography (CESM) - is it as sensitive as breast MRI in the detection of breast lesions?

### Elizabeth Lepine-Williams

#### Guys and St Thomas NHS Foundation Trust, London, UK

**Introduction**

CESM produces low and high energy images at specific time intervals taken once a patient has been injected with an IV contrast agent. In the post processing phase the two images are recombined producing an image in which contrast enhancement is visible. Studies show it to be as sensitive as MRI in detecting uni and multi focal disease, potentially providing an alternative to MRI for specific patient groups.

**Methods**

A single centre matched study comparing the sensitivity and specificity of CESM with breast MRI of patients who had both CESM and MRI performed within the trust was conducted. Images were reviewed and the following data extracted:

Number of lesions

Lesion size

Histology results - grading and size

Data was analysed; sensitivity, specificity, PPV and NPV of CESM verses MRI were calculated.

**Results**

A total of 31 women participated in the study.

Results show that CESM had a sensitivity of 97% (36/37) and a specificity of 60% (3/5) with a PPV of 94% (36/38) and a NPV of 75% (4/5). MRI had a sensitivity of 100% (38/38) and a specificity of 75% (3/4) with a PPV of 97% and a NPV of 100%.

**Conclusion**

Results show the sensitivity and specificity of CESM is similar to that of MRI. Preliminary findings show, in specific cases CESM could be used as an alternative to MRI in lesion detection. As this is a small sample size, further analyses of future data should be performed to see if changes to practice can be implemented.

## PB.29 Image versus imaging: Do breast implants compromise cancer detection at screening?

### Steven Henderson^1,2^, Janet Litherland^1,2^

#### ^1^NHS Greater Glasgow & Clyde, Glasgow, UK; ^2^West of Scotland Breast Screening Programme, Glasgow, UK

##### **Correspondence:** Steven Henderson

**Introduction**

There is ongoing concern that silicone implants, particularly those placed in a subglandular position, may reduce the sensitivity of mammography in the detection of malignant lesions. Our aim was to determine whether or not breast implants affected the cancer detection rate in our screening population.

**Methods**

A retrospective search was undertaken to identify all patients with breast implants who had been diagnosed with breast malignancy, through the West of Scotland Breast Screening Programme (WOSBSP), over a period from April 2010 - March 2015. The number of malignancies was then compared with the number detected in the non-implant population during the same time period.

**Results**

During the period, 310,558 patients attended for screening. Of these, 852 had breast implants.

A total of 2,840 malignancies were detected, 6 of which were in patients with implants - 1 prevalent and 5 incident. All 6 had subglandular implants. There were 3 diagnoses of DCIS and 3 of invasive carcinoma, with a size range of 10-34mm. None of the patients had nodal disease at the time of diagnosis.

The malignancy detection rate in the implant group was 7 per 1000 and 9.2 per 1000 in the non-implant group (P=0.52).

**Conclusion**

Although there appears to be a trend towards a lower cancer detection rate in women with implants, the results do not reach statistical significance.

However our study is limited by small numbers of cancers in women with implants and this also precludes further analysis of data for invasive and small cancer detection rates.

## PB.30 Correlation of radiological and pathological findings in breast implants – how accurate are imaging appearances?

### Naveed Altaf, Yvonne Bury, Nerys Forester

#### RVI, Newcastle, UK

##### **Correspondence:** Naveed Altaf

Imaging of breast implants is increasingly performed to investigate suspected leak or rupture. However, there is little opportunity for the radiological findings to be correlated with the pathological findings, providing feedback as to the accuracy of the radiological diagnosis. We reviewed pathological findings in explanted breast implants, in conjunction with the radiological findings, to investigate the accuracy of imaging for breast implant changes.

The pathology database was searched for all breast implants removed between July 2000 and January 2015. All patients identified had their imaging history reviewed to identify those with pre-operative imaging. Pathological findings at implant removal and radiological findings on the pre-operative imaging were compared.

There were 106 implant related pathology cases identified. From this, 11 patients had explant of breast implants and pre-operative imaging. In 8 cases implants had been placed for cosmetic augmentation, 3 were following implant-based reconstruction. 10 cases had pre operative ultrasound, 1 had MRI and 1 had both. Radiological findings demonstrated intra or extra capsular rupture in 9, silicone granulomas in 1 and an intact implant in 1 case. Imaging findings were consistent with the pathological findings in all cases.

Imaging of implants provides an accurate pre-operative assessment of implant pathology. As many more implants are imaged than removed, and many implants removed without any pre operative imaging, it is difficult to get feedback about the accuracy of radiological diagnoses in this setting. Although small, this study shows 100% concordance between radiological and pathological findings following the pre-operative imaging of implant pathology.

## PB.31 Correlation of symptoms with breast implant rupture

### Mei Chan Chin, Janet Litherland

#### NHS GGC, Glasgow, UK

##### **Correspondence:** Mei Chan Chin

**Introduction**

Patient’s symptoms and clinical examination are useful but can be non-specific for breast implant rupture. We wanted to determine in our unit how the presenting clinical features correlated with implant rupture diagnosed on MRI.

**Methods**

A CRIS search was done for patients who had breast implant protocols performed at our unit from 15/07/2015 dating back to 12/12/2013. Data was collected from CRIS and Clinical Portal on symptoms and implants.

Symptoms were grouped into three categories:

(A) Pain/tenderness/discomfort
(B) Texture/shape/size/volume change
(C) Palpable/lump

Implant information collected included age, filling (saline/silicon/Becker), lumen (single/double), position (subglandular/subpectoral).

**Results**

Forty eight patients had MRI leading to assessment of 89 implants. Sixteen implants were ruptured, in 14 patients. 14(87.5%) presented with pain; 9 (56.3%) had category (B) symptoms; 5 (31.1%) had a lump.

Mean ruptured implant age was 16.9 years. Interestingly, these implants were in situ >10 years apart from 4. One of these developed symptoms after assault whilst the remainder were PIP implants. Once these were excluded, mean age was 20.8 years.

All ruptured implants were silicon except for one saline implant.

Positive predictive values (PPVs) for rupture were 40% for pain, shape 25.7%, lump 31.3%, implant age >10 years 37%, single lumen 18.8% and silicon 17.8%.

**Conclusion**

Pain and implant age >10 years have the highest PPVs for rupture at 40% and 37% respectively. Overall, however, no symptoms or implant factors were specific for rupture.

It is less likely that an implant <10 years would be ruptured unless in exceptional circumstances eg trauma/PIP.

## PB.32 A retrospective review of recalls from an NHSBSP high risk MRI screening programme

### Caroline Taylor, Pippa Skippage, Aneet Sian, Rita McAvinchey

#### Jarvis Breast Screening Centre, Guildford, UK

##### **Correspondence:** Caroline Taylor

Annual high risk screening MRIs have been reported at the Jarvis Breast Screening unit since January 2014. There are currently 182 patients on our database which include patients with BRCA 1 or 2 mutation and patients who have received mantle radiotherapy below the age of 30.

In 2014: 17 patients were recalled for further assessment. In 2015: 23 patients were recalled. Four Consultant Radiologists who also report symptomatic MRI's took part in double reading.

Following the introduction of this new service we felt it was important to review our practise. This was done in order to understand our recall rates and review the findings at assessment including results of ultrasound and MRI guided biopsies, cancer detection, early recall and interval cancer rates. We include a pictorial review of examples of the recalled lesions.

## PB.33 A 15 year review of familial breast cancer screening in Wales - are we offering the right test to the right women?

### Tom Evans^2^, Guy Stevens^1^, Sian Nisbet^3^, Alex Murray^3^, Kate Gower Thomas^1^

#### ^1^Breast Test Wales, Cardiff, UK; ^2^Royal Gwent Hospital, Newport, UK; ^3^All Wales Cancer Genetics Service, Cardiff, UK

##### **Correspondence:** Tom Evans

**Introduction**

Since 2001, the Welsh breast cancer screening programme has offered annual mammography to women below age 50, identified as having an increased risk of the disease. Women are referred via the All Wales Genetics Service who analyse client risk and apportion a risk category. Women are screened from either aged 35 or 40 years accordingly. We present results including cancer detection rate, numbers of screening eposodes and try to determine whether this is cost effective.

**Method**

A prospectively maintained NHSBSP equivalent database for women with a significant family history of breast cancer was analysed retrospectively for uptake of screening and cancer detection, including interval cancer episodes. The costs of this service were estimated.

**Results**

5586 moderate and high risk women have been invited for annual screening. The average prevalent uptake was 82.4% and a total of 22270 mammograms were performed. 118 cancers were detected, 84 were screen detected and 34 were interval cancers. The cancer detection rate in this moderate and high risk population combined was 3.8/1000 screenings, which is significantly lower than 10/1000 seen in population screening (p<0.05). Cancer detection in these moderate and high risk women was only a third of the general population suggesting this screening technique is inappropriate and should be abandoned for reasons of cost, stress and unnecessary radiation exposure. We suggest that other screening methods, with further risk stratification might be more effective.

## PB.34 Management and future risk of malignancy in patients diagnosed with Lobular Neoplasia In Situ (LNIS)

### Raquel Clark Castillo^1^, Hannah Markham^1,2^, Rachel Oeppen^2^

#### ^1^University of Southampton, Southampton, UK; ^2^University Hospital Southampton, Southampton, UK

##### **Correspondence:** Raquel Clark Castillo

Lobular neoplasia in situ is classified as a B3 lesion of uncertain malignant potential and confers an increased risk of concurrent and future malignancy. In the absence of national guidance there is uncertainty over optimal management and peak malignancy risk may not fall within the 5 years of annual surveillance recommended by some centres.

Biopsy reports from 1999-2015 at Southampton General Hospital were screened for code B3 and/or diagnosis of LNIS with collection of a comparison group of patients with B2 diagnoses. Recommended management was noted and screening packets were reviewed to identify subsequent assessments and biopsies.

LNIS was diagnosed in 130/12,141 breast biopsies (1.07%) performed between 1999-2015 Concurrent malignancy was present in 44/130 (34.68%) of which 33/44 were infiltrative lobular cancer and a further 7/130 (0.05%) had a B4 or B5a diagnosis. Management of pure B3 lesions was highly varied in those with available follow-up data (58/130) but most commonly comprised routine recall following surgical excision biopsy (34.5%). Women with a B3 diagnosis of LNIS were at increased risk of developing invasive malignancy compared to the B2 group (8.62% and 3.13% respectively, p=0.21) and the average time to malignancy was 15 years.

The high rate of concurrent malignancy, in particular invasive lobular cancer, may support the theory that LCIS is a precursor lesion. LCIS also confers an increased risk of subsequent invasive neoplasia which in this study occurred on average 15 years from LCIS diagnosis. This timeframe should be considered when recommending management with enhanced mammographic surveillance.

## PB.35 The changing risk factor profile of Asian women with breast cancer

### Timothy Iype Thomas^1^, Anil Jain^1,3^, Jaanilka Molderson^1^, Joseph Wan^2^

#### ^1^The Nightingale Centre and Genesis Prevention Centre, University Hospital of South Manchester, Manchester, UK; ^2^The University of Manchester, Manchester, UK; ^3^Manchester Academic Health Sciences Centre, Institute of Cancer Sciences, University of Manchester, Manchester, UK

##### **Correspondence:** Timothy Thomas

**Aim**

To investigate the changes in risk factor profile across time in Asian women with breast cancer.

**Method**

321 Asian patients diagnosed with breast cancer at the Nightingale Centre and Genesis Prevention Centre, University Hospital South Manchester between 1999 to 2016 were divided into 6 year groups according to their year of diagnosis. Established breast cancer risk factors were analysed across the three groups to identify potential changes and trends. Statistical analysis was performed across these three groups primarily using analysis of variance (ANOVA) for continuous variables and Pearson’s chi-squared tests for categorical variables.

**Results**

There was a decreasing trend of proportion of patients with diabetes mellitus (p<0.001). There were increasing trends for mean age of first full term pregnancy, mean duration of interval between menarche and first full term pregnancy, proportion of women who breastfeed, and BMI. There was a decreasing trend of proportion of multiparous women. However these findings were statistically insignificant.

**Conclusions**

There are changing trends in certain risk factors for breast cancer which might contribute to the increasing incidence of breast cancer in Asian women. More research has to be done on modifiable breast cancer risk factors and how they can be altered to decrease breast cancer risk.

## PB.36 A survey of radiographer film reader's perceptions of workload, performance and job satisfaction in the NHSBSP

### Helen Newman

#### Chelmsford & Colchester Breast Screening Department, Essex, UK

A survey of radiographer film reader's perceptions of workload, performance and job satisfaction in the NHSBSP.

**Introduction**

Age extension has increased the film reading workload in breast screening. A reported shortage of radiologists plus radiographers double reporting also has the potential to increase reading volumes. There is disagreement on whether performance declines with increasing volumes. There are no recommendations on maximum reporting volumes. This survey aims to identify themes which affect film readers’ perceptions of workload, performance and job satisfaction.

**Methods**

Purposeful sampling was used to select participants. All qualified radiographer film readers were included. Electronic questionnaires were distributed to managers to forward to participants. Thematic analysis was used to analyse results.

**Results**

The overall response rate was 37%. 77% perceived an increased workload. 60% report a sufficient workforce for reporting. 40% report high volumes. Performance is thought to fluctuate following interruptions, PERFORMS and fatigue. 84% are satisfied in their role.

**Conclusion**

Radiographers are experiencing an increase in workload but reporting time is frequently interrupted. Audit should assess any effect on performance by high volumes and visual fatigue. More time for CPD is required and increased involvement in research and audit. Job satisfaction is high amongst readers which should aid retention and recruitment. With a potential future shortfall in radiologists, the NHSBSP will be reliant on advanced and consultant practitioners to maintain targets. There is the potential to further develop the role but care should be taken not to compromise performance and job satisfaction with the volume of work.

## PB.37 Time to actual assessment: A true performance indicator?

### Simon Lowes, Linsley Lunt

#### Breast Screening Unit, Queen Elizabeth Hospital, Gateshead, UK

##### **Correspondence:** Simon Lowes

**Introduction**

The NHSBSP KPIs require 90% of first offered appointment (FOA) *and* first actual assessment (AA) to be within 21 days of screening mammography. This leaves little flexibility for women unable to attend FOA. Our unit consistently achieves FOA (monthly rates 93%-100% in last round), but frequently breaches the AA (monthly rates 78%-100%). We investigated why the breaches occur.

**Methods**

NBSS data were analysed for women recalled from screening for the 3 years spanning April 2013-March 2016.

**Results**

Of 3595 women recalled to assessment, 99.6% of FOA were within the required timescale. Of these, 464 women were not able to make their FOA. 113 women made an alternative appointment and attended assessment within the 21 day limit; the remaining 351 did not.

Overall, 377 (10.5%) of recalled women failed to have their AA within 21 days, with seasonal variation observed. In just 3.7% of all breaches was the unit unable to make a FOA within 21 days. A further 5.0% comprised a data entry error on NBSS, erroneously registering as a breach.

Of the remaining breaches, 76.7% changed appointment at least once; 14.6% failed to attend FOA (DNA) without contacting the unit; 1.1% had a DNA and made at least one other appointment change. Reasons women changed appointments: inconvenient 55.0%; on holiday 37.4%; unwell 3.1%; combination of these 4.5%.

**Conclusion**

Time to actual assessment frequently reflects client choice and/or circumstances. These factors are outside the control of the unit, making the time to actual assessment a questionable measure of performance.

## PB.39 Outcome of assessment of patient reported symptoms during screening mammograms - Single NHS BSP unit's experience

### Sadia Sultana, Varun Chillal, Muthyala Sreenivas

#### University Hospital Coventry and Warwickshire, Coventry, UK

##### **Correspondence:** Sadia Sultana

Patients are advised to report any breast associated symptoms during their screening mammograms in the Warwickshire, Solihull and Coventry Breast Screening Service (based at UHCW NHS trust). Although no specific guidance on whom to recall exists, symptoms such as lump, distortion/change in shape of breast, recent nipple discharge, eczema, or recent inversion, skin tethering and dimpling are routinely recalled for further assessment. In this retrospective study, we investigated the types of symptoms reported and subsequent clinical, imaging and histology findings with specific aim of finding the number of cancers in this cohort of patients.

Between 2011 -2015 data was collected from NBBS and hospital RIS, PACS and CRRS on patients reporting symptoms during their routine screening mammogram. This included 709 patients (age range: 47 to77 years, mean age of 56 years).

Subsequently, 15 patients were diagnosed with breast cancer, of these 3 patients were excluded as 2 of them were originally referred by the radiographers who noticed the changes at the time of screening and the third patient would have been recalled anyway for abnormal mammography.

Among the remaining 12 patients, 6 had cancer on a site or side completely different from the actual location of symptom patient identified.

Therefore, 6 out of 709 patients (0.84%) who were recalled were concordant with clinical symptoms.

This study shows the positive predictive value (PPV) of patients who reported symptoms turning into a breast cancer is low. Therefore, an alternate pathway of managing these patients should be investigated.

## PB.40 The association between regional disposable household income and uptake of breast screening in England between 1999 and 2014

### Rebecca Dalton^1^, David Reeves^2^, Jamie Sergeant^3,4^, Elaine Harkness^5,6^, Soujanya Gadde^6^, Anthony Maxwell^5,6^, Yit Lim^6^, Susan Astley^5,6^

#### ^1^Manchester Medical School, University of Manchester, Manchester, UK; ^2^Centre for Primary Care, University of Manchester, Manchester, UK; ^3^Arthritis Research UK Centre for Epidemiology, Centre for Musculoskeletal Research, Manchester Academic Health Science Centre, The University of Manchester, Manchester, UK; ^4^NIHR Manchester Musculoskeletal Biomedical Research Unit, Central Manchester University Hospitals NHS Foundation Trust, Manchester, UK; ^5^Division of Informatics, Imaging and Data Sciences, School of Health Sciences, University of Manchester, Manchester, UK; ^6^Nightingale Centre and Genesis Prevention Centre, University Hospital of South Manchester, Manchester, UK

##### **Correspondence:** Rebecca Dalton

**Aim**

To examine possible associations between regional disposable household income and uptake of breast screening and investigate whether any association is related to the type of invitation, categorized as: first invitation; invitation to previous non-attender; invitation to previous attender (last attendance <5 years); invitation to previous attender (last attendance >5 years) and early recall invitation.

**Methods**

Data on breast screening uptake and Gross Disposable Household Income (GDHI) per head for the English regions for the years 1999-2014 were obtained from the Health and Social Care Information Centre and the Office for National Statistics respectively. Uptake data were adjusted to fit calendar year and GDHI data were adjusted for inflation. The association between these was assessed using multiple linear regression.

**Results**

There was a significant (p<0.05) positive association between regional GDHI per head and overall breast screening uptake with a coefficient of 0.00066 (95% confidence intervals 0.00027- 0.00106). This is equivalent to a 0.66% rise in breast screening uptake for every £1000 increase in regional GDHI per head. No type of invitation alone showed a statistically significant association between GDHI per head and breast screening uptake.

**Conclusion**

Regional disposable household income has a positive association with uptake of breast screening in the English regions. In this study, there was no significant association between GDHI per head and breast screening uptake for any one type of invitation. Low income populations should be targeted to reduce inequalities and further research should determine which interventions can be cost effectively applied to these populations.

## PB.41 Is double reading still a requirement in the NHSBSP in the digital era?

### Liz Edwards^1,2^, Kate Jenkins^1,2^, Guy Stevens^2^

#### ^1^Breast Test Wales, South East Wales, UK; ^2^Public Health Wales, Wales, UK

##### **Correspondence:** Liz Edwards

**Introduction**

There is a current workforce crisis in breast radiology. This review determines if double blind double reading continues to be necessary as since the introduction of digital mammography individual cancer detection rates have increased. Are single reader recalls contributing to the detection of pathologically insignificant cancers.

**Method**

This is a retrospective analysis of prospectively collected data from the NBSS database at BTW (S. E.). The readers had experience from trainee to 20 years+. All single reader recalls that were assessed and diagnosed as cancer were included from 2010-2015. Pathological data was collected including size, tumour type, grade and node positivity. Data pre and post digital screening mammography were evaluated.

**Results**

450 women were identified for analysis as single reader recalls. Of the single reader recall cases, 317 were invasive and 131 non -invasive. The majority (83.5%) of malignancies detected were TMN stage 1(Average size 14mm, mode 8mm, range 1-150mm). Of the invasive malignancies identified, 103 were grade 1, 172 were grade 2 and 39 were grade 3 at final pathology. The total number of patients that were node negative at subsequent surgery was 91.33%. 14.4% of all cancers detected were attributed to a single reader recall. All readers contribute to the single recall rate regardless of reader status.

**Conclusion**

Single reader recall detected cancers make up a significant proportion of the cancers detected and are pathologically significant. It remains important to continue with double blind reading despite workforce issues and despite individually good cancer detection rates.

## PB.42 Improving breast screening communication with South Asian (SA) women: Usage of multilingual videos on a handheld device

### Anil Jain^1,2^, Jaanilka Molderson^1^, Shaheeda Shaikh^1^

#### ^1^The Nightingale Centre and Genesis Prevention Centre, University Hospital of South Manchester, Manchester, UK; ^2^Manchester Academic Health Sciences Centre, Institute of Cancer Sciences, University of Manchester, Manchester, UK

##### **Correspondence:** Anil Jain

**Background**

The South Asian population has increased across the UK along with increase in breast cancer incidence in them. Yet the uptake rate for breast screening in this group of women remains significantly lower. Barriers to screening, such as language barriers can deter some women from attending.

**Materials and Methods**

A pilot questionnaire study was carried out to assess the screening experience of 110 women who watched a breast screening video on a tablet device in their choice of language prior to screening. The screening experience was assessed by a five item questionnaire. For advanced understanding of the device and its related issues, supplementary data was collected in the form of a radiographers’ feedback questionnaire (n=92).

**Results**

The majority of the women participating were supportive towards this method of help (85%) and had favourable opinion about the device (90%). In total 93% of the women agreed that the video helped them through the mammography procedure and 79.2% agreed that it made their screening experience better. The radiographers’ feedback suggested that time was a sensitive issue in applying this method, with 39% suggesting it increased the clinic times.

**Conclusion**

The usage of multilingual tablet device was perceived positively by the majority of women attending for breast screening. It was considered helpful in improving the individual’s screening experience and possibly will have a positive impact on their compliance in subsequent screening rounds. The tablet device should be considered for national piloting as part of the National Breast Screening Programme.

## PB.43 A review of MRI Guided Breast Interventions at the Royal Marsden Hospital

### Richard Sidebottom^1,2^, Elizabeth O’Flynn^1^, Laura Bombroffe^1^, Julie Hughes^1^, Erica Scurr^1^, Robin Wilson^1^

#### ^1^Royal Marsden NHS Foundation Trust, London, UK; ^2^Oxford University Hospitals NHS Foundation Trust, Oxford, UK

##### **Correspondence:** Richard Sidebottom

**Background**

MRI guided breast biopsy is required to assess MRI abnormalities not demonstrated on conventional imaging.

**Methods**

We performed a retrospective audit of all MRI guided breast interventions at the Royal Marsden from 2012, when the service began, to 2015. Data was obtained the Electronic Patient Record and MRI records. MR imaging characteristics and histology were recorded along with the types of referrals, technical aspects of the procedures and complications.

**Results**

57 patients were booked for an MRI guided intervention (31 Royal Marsden patients, 26 external referrals). The appointment for biopsy was on average 14.5 days after the MDT decision. The average procedure duration was 89 minutes.

No enhancing abnormality was demonstrated on procedural MRI in six cases, other technical reasons preventing successful biopsy included posterior positioning of the lesion (n=1) and software failure (n=1, successfully repeated).

Of 47 biopsies performed, histology showed 15(32%) B4/B5a/B5b, 4(9%) B3 and 28(60%) B1/B2. In situ disease more commonly demonstrated non-mass like enhancement (3/4) whereas invasive disease usually demonstrated mass features (9/10).

There were 4 ‘significant bleeds’ requiring monitoring in the department and one vasovagal collapse which necessitated repeat procedure.

**Conclusions**

Our cancer detection rate (32%) is comparable to other UK centres^1^ and higher than studies in Europe (21%) and USA (8%)^2,3^, suggesting appropriate case choice. Technical difficulties and complications were part of the learning curve, and practices have evolved to minimize these.

1.Teh et al. Br Can Res Supl 2012;1:03

2.Spick et al. Eur Rad 2016epub

*3.Myers et al. 2015;15(2):143-152*

## PB.44 Initial single centre experience with the Intact^TM^ percutaneous breast lesion excision system (*Intact*) using Ultrasound (USS) Guidance

### Lyn Jones, Sasirekha Govindarajulu, Ajay K Sahu

#### Bristol Breast Care Centre, North Bristol NHS Trust, Bristol, UK

##### **Correspondence:** Lyn Jones

**Introduction**

***Intact*** is a breast biopsy device that excises a breast abnormality using vacuum and radiofrequency (RF) technology. It is licenced in UK as a mammographic-mounted device, or a hand held device for USS guidance. Research from USA showed complete removal of breast abnormalities occurs frequently during ***Intact*** breast biopsy but experience in UK is limited. We present our centre’s initial experience using ***Intact*** under USS guidance.

**Methods**

Selection of masses for ***Intact*** depended upon the size of mass and the willingness of the patient to undergo the procedure, initially under general anaesthesia (GA) immediately prior to her therapeutic excision, and subsequently under local anaesthetic (LA). For the local anaesthetic cases an additional criteria for U5 masses was that surgical excision under general anaesthetic was contraindicated for the patient due to co-morbidity.

**Results**

- 19 selected breast masses in 15 women were biopsied under USS guidance using “Intact”.
- GA: 11 masses in 9 women (January 2012–July 2013).
- LA: 8 masses in 6 women (September 2015-July 2016).
- Histology included B2 (8), B3 (4) and B5 (7) masses.
- Sizes for B3 and B5 masses: 4-17mm.
- 6/7 B5 and 4/4 B3 masses - histology confirmed complete excision with Intact.
- No complications from the ***Intact*** procedures.

**Conclusion**

Our initial experience suggests it is safe to perform ***Intact*** under LA and USS guidance in an outpatient setting and that Intact offers the potential to avoid subsequent excisional surgery for small B3 and carefully selected small B5 masses in the future.

## PB.45 Differences in acute and persistent pain following ultrasound and stereotactic guided vacuum-assisted breast biopsy (VABB) - results of a pilot survey

### Matthew Brown^1,2^, Steve Allen^1^, Liz O’Flynn^1,2^, Nandita deSouza^1,2^

#### ^1^The Royal Marsden Hospital, London, UK; ^2^The Institute of Cancer Research, London, UK

##### **Correspondence:** Matthew Brown

**Purpose/background/objectives**

Vacuum assisted breast biopsy (VABB) is a minimally-invasive modality enabling target lesions identified within breast tissue to be either sampled or removed. A biopsy needle is advanced percutaneously to the target under stereotactic, ultrasound or MRI guidance, whence multiple samples are harvested. This pilot survey explored whether differences in acute and persistent pain intensity occurred between ultrasound and stereotactic-guided VABBs.

**Methods**

A questionnaire-based survey was undertaken; basic demographic and procedural data for patients was recorded at the time of VABB. Participants completed a pain/analgesia diary detailing the intensity of pain experienced and analgesia taken over the 7 day post-VABB period. Participants were contacted at 3 months post-VABB to determine the presence of persistent pain.

**Results**

49 participants were recruited and 38 completed questionnaires were returned (27 US, 11 stereo’). Statistically significant differences were observed in the intensity of pain experienced post-biopsy by patients who underwent US and stereo VABB on day 1; 3.4 (SD 2.8) vs 1.4 (SD 1.6) *P*=0.04, day 4; 1.3 (SD1.5) vs 0.2 (SD 0.6) *P*=0.03, day 6; 0.7 (SD 0.9) vs 0 *P*=0.03 and day 7; 0.7 (SD 1.0) vs 0 *P*=0.03. No procedural differences existed between the groups. 3 patients (8%) reported persistent pain at the 3-month time point, all had undergone US-guided VABB.

**Conclusions**

Patients undergoing US guided VABB experienced more intense pain in the week following biopsy than those undergoing sterotactic guided VABB and appeared to experience more persistent pain. Further work is required to determine the cause of these findings.

## PB.46 An audit of new biopsy technique

### Rumana Rahim, Rema Wasan, Jane Goligher, Shalini Wijesuriya, Juliet Morel, Clare Peacock, Michael Michell, Asif Iqbal, David Evans, Bhavna Batohi, Tavleen Wasan, Vivien Phillips, Eden Manuel, Gillian Bowman, Keshthra Satchithananda

#### Kings College Hospital, London, UK

##### **Correspondence:** Rumana Rahim

**Aim**

Largest series comparing the unit current standard-of-care, Prone-stereo(PS) Vacuum Breast Biopsy(VAB) with Digital Breast Tomosynthesis (DBT)-guided VAB.

**Background**

DBT is an established mammographic technique shown to improve the accuracy of soft tissue lesion characterisation, conspicuity and cancer detection. VAB increases the sensitivity of pre-operative diagnosis, reducing upgrade rates.

**Method**

170 consecutive patients through the NHSBSP and symptomatic breast service undergoing stereo-guided biopsy were recruited into 2 arms:

Multicare-Platinum-Prone-Stereotactic-Breast-Biopsy-System

Affirm-Breast- Biopsy-Guidance-System with 3D Breast Biopsy using Hologic Selenia Dimensions

VAB was performed using the Atec-Sapphire with 9G Eviva needles. Procedure outcomes including patient comfort, time, accuracy, radiation dose and complications were recorded.

**Results**

85 patients in each arm.

DBT-guided lesions: Microcalcification 72, Mass 4, Distortion 9

PS-guided lesions: Microcalcification 82, Mass 3, Distortion 0.

Benefits of DBT compared with PS VAB were:

- Shorter: Mean Room-Time 35 minutes versus 43 minutes, Mean Compression-Time 19 minutes versus 23 minutes
- Accuracy: 97.6% versus 94%
- Lower mean total radiation dose: 14.83mGy versus 39.4mGy
- Fewer mean exposures: 7 versus 10

In addition:

- Patient pain, comfort and acceptability scores were comparable as were operator ease of use scores
- 4% required prolonged compression for haemostasis with DBT compared with 2.5% with PS
- 6% vasovagal-rate with upright DBT compared to 0% with decubitus DBT or PS.

**Conclusion**

We demonstrate DBT-guided biopsy is a good alternative technique to stereo-guided biopsy for soft tissue abnormalities (including those occult on 2D and ultrasound) and microcalcifications. There is an increased risk of vasovagal episodes with upright DBT, which could be avoided with decubitus positioning or Prone-DBT.

## PB.47 10-year review of screen-detected lesions of uncertain malignant potential (B3) - How has our practice changed

### Bhavna Batohi, Cheng Fang, Michael Michell, Juliet Morel, Chirag Shah, Shalini Wijesuriya, Clare Peacock, Rumana Rahim, Rema Wasan, Jane Goligher, Keshthra Satchithananda

#### King’s College Hospital, London, UK

##### **Correspondence:** Bhavna Batohi

**Introduction**

The management of B3 lesions is becoming increasingly under debate in light of criticism of over-diagnosis within breast screening. New proposed guidelines throughout the UK are suggesting that surgical biopsy for many B3 lesions may no longer be required.

In this audit we review all cases of B3 at initial biopsy over two five-year cohorts.

**Methods**

Data was collected using the National Breast Screening System.

**Results**

There were 224 cases in 2005-2010 and 240 cases in 2010-2015.

Mammographically 211 lesions were microcalcifications, 182 masses, 65 distortions and 6 asymmetries.

208 14G core biopsies and 256 initial vacuum biopsies were performed.

50% of patients in the first cohort underwent benign surgical biopsy compared to 40.4% in the latter cohort.

There was a 6% upgrade to invasive malignancy and 18% upgrade to non-invasive malignancy over the 10-year period following surgical biopsy and vacuum excisions.

The upgrade rates for each histological category were:

- Atypical duct hyperplasia 36%
- Flat epithelial atypia 28%
- Radial scar/complex sclerosing lesion with atypia 27% and with no atypia 11%
- Papilloma with atypia 55% and with no atypia 16%
- Lobular neoplasia in situ 43%
- Suspected phyllodes tumours 8%
- Atypical apocrine adenosis 20%.

**Conclusions**

The results of this audit and upgrade rates are in line with the literature. Upgrade rates remain high even with first line use of vacuum biopsy. Careful consideration is essential prior to changing current practice.

## PB.48 Prospective follow up of patients with B3 lesions over a 4-year period

### Naveed Altaf, Nerys Forester

#### RVI, Newcastle, UK

##### **Correspondence:** Naveed Altaf

Following introduction of large-bore vacuum-assisted biopsy (LVB) for diagnosis and management of B3 lesions in 2011, a prospective database of patients was developed. Following B3 lesion diagnosis on core biopsy, patients underwent LVB. If B5, patients had surgery; if B3 patients underwent 5 years annual surveillance mammography (ASM) or were discharged/returned to routine recall, depending on the presence of epithelial atypia. Outcomes were prospectively audited over 4 years.

B3 lesion database analysed to ascertain number of ASMs performed, recall rate, symptomatic episodes and subsequent malignancy following B3 diagnosis.

Between October 2011 and December 2015, 396 patients had a B3 lesion. 305 underwent second line LVB. 27 patients diagnosed with malignancy following LVB and 17 patients were upgraded to malignancy following excision biopsy (unsuitable for LVB/pathology request).

352 patients had ASM/routine recall, together having 410 mammograms performed over 4 years. 9 patients were recalled from ASM (recall rate 2%). 20 underwent further breast investigations (19 presented symptomatically, 1 recalled from MRI high-risk surveillance).

From additional investigations 3 cancers diagnosed (1 following high-risk surveillance MRI; 1 symptomatic presentation, 1 recall from ASM). There were also 4 further B3 lesions and 22 benign diagnoses.

LVB for B3 lesions is an excellent alternative to excision biopsy. However, ASM has a low recall rate and cancer detection rate, with only 1 of the 3 subsequent cancers detected by mammographic surveillance. This questions whether ASM is really necessary in this group of patients. Could they be safely returned to routine recall within the screening program?

## PB.49 Inflammatory breast cancers (IBC)

### Leanne Hodkin, Keiran Horgan, David Dodwell, Nisha Sharma

#### Leeds Teaching Hospitals Trust, Leeds, UK

##### **Correspondence:** Leanne Hodkin

**Introduction**

IBC is an extremely aggressive breast cancer with a very poor prognosis. Routine imaging includes staging CT and bone scan. PET CT is considered superior to CT. We reviewed cases of IBC in Leeds from 2008-2014 to determine the radiological investigations performed and patient outcome.

**Methods**

From 1^st^ February 2008 – 31^st^ September 2014 all IBCs managed in Leeds were retrospectively identified. The radiological investigations, histology, subsequent management and outcome were documented.

**Results**

40 patients were included, 28 had an initial staging CT and 12 did not. 4/28 (14%) had metastatic disease on initial staging CT. 19/28 (68%) had a negative staging CT and 5/28 (18%) had indeterminate CTs due to the presence of lung nodules/liver lesions.

Of the initial 19 negative staging CT scans, 1 patient died of an unrelated condition. 9/18 (50%) developed metastatic disease at follow up *(6months-5years).* Of the indeterminate CTs, 2/5 (40%) went on to develop metastases at 2 months and 2 years respectively.

**Conclusion**

Metastatic disease is common in women presenting with IBC and accurate staging is essential in guiding management and providing prognostic information to patients. 70% of our cohort had an initial staging CT, when all patients should be undergoing this. Of those who received an initial negative staging scan, 32% of these had metastatic disease within 12 months. In view of this, a PET/CT is the recommended staging modality of choice to allow for more accurate staging in this cohort,enabling better management in this select group of patients.

## PB.50 The potential use of Photoacoustic Imaging in Breast Cancer

### BM Moloney^1^, MC Casey^1^, A Breathnach^2^, RM Dwyer^1^, R McLoughlin^1^, KJ Sweeney^1^, C Malone^1^, MJ Healy^2^, MJ Kerin^1^

#### ^1^Department of Surgery, National University of Ireland, Galway, Ireland; ^2^Department of Physics, National University of Ireland, Galway, Ireland

##### **Correspondence:** BM Moloney

**Introduction**

Breast cancer mortality is intrinsically linked to stage at diagnosis, so the ability to detect disease in infancy is critical. Sub optimal sensitivity and specificity of current imaging methods such as mammography and ultrasonography result in issues such as false positive and false negative results. Photoacoustic Imaging (PAI) represents a novel approach to breast tissue visualisation. Non-ionizing laser pulses are delivered to tissue and photoacoustic signals generated. Coupled with ultrasound emission, structural imaging and functional analysis of tissue can be determined. This pilot study assesses the potential use of the VisualSonics Vevo LAZR PAI system in the investigation of normal and abnormal breast tissue.

**Methods**

Ethical approval and informed consent was obtained. Using a 15MHz probe, imaging of healthy and abnormal breast tissue was performed in a tertiary symptomatic breast cancer unit (n=8). Oxygen saturation, haemoglobin concentration and photoacoustic signal was determined for all.

**Results**

Characterisation of healthy breast tissue, tissue with benign pathology (fibroadenoma) and tissue with previously identified breast cancer was achieved. With locations of interest determined by ultrasound, photoacoustic signals were assessed. Imaging to a depth of 30mm was confirmed. Comparisons in oxygen saturation were made between breast pathology and the controls (disease free contralateral breast).

**Conclusion**

Current imaging modalities in breast cancer have shortcomings. PAI represents a novel means of both visualisation and assessment of functionality of breast tissue. These preliminary findings offer an insight into the potential of PAI in the role of breast cancer diagnosis.

## PB.51 Systematic review of breast lesions of uncertain malignant potential (B3 lesions) and their risk of malignancy

### Nerys Forester^1^, Simon Lowes^1^, Elizabeth Mitchell^2^, Maureen Twiddy^2^

#### ^1^RVI, Newcastle, UK; ^2^Institute of Health Sciences, Leeds University, UK

##### **Correspondence:** Nerys Forester

Borderline breast lesions (B3 lesions) can coexist with malignancy. The magnitude of this risk varies between studies and lesion subtypes. This systematic review of the literature will determine an accurate estimate of the risk of invasive/in-situ malignancy identified by surgical excision biopsy, following diagnosis of a B3 lesion at core biopsy, within each B3 lesion subtype, to guide risk stratification and improve management strategies.

Literature searches (MEDLINE, Embase, HMIC, Scopus and Web of Knowledge), identified relevant studies between 1980 and 2014. Literature appraisal, meta-analysis and subgroup analysis performed to determine malignancy risk for all subgroups of B3 lesions (Papilloma, Radial Scar, AIDP, Lobular Neoplasia and FEA).

Searches returned 2289 citations, with 11 identified from other sources. Duplicate, unsuitable articles and abstracts/posters/reviews were excluded leaving 183 records. From these, 54 full text articles did not meet inclusion criteria. Meta-analysis was performed from 129 studies. Rates of malignancy varied from 6% in a radial scar with no atypia, to 32% for a papilloma with atypia. Differences in malignancy upgrade rates between atypical and non-atypical lesions were statistically significant (p<0.05). Study heterogeneity could not be explained by differences in core biopsy size or year of publication, however, a significant difference in upgrade rates to malignancy was observed between the US and non US literature.

Many studies have assessed the risk of malignancy following diagnosis of B3 lesions, but are often small and lack statistical power. This study is a comprehensive, inclusive assessment of the available literature, on which to base tailored management strategies.

## PB.52 The use of supplemental imaging in post-surgical follow-up of women with breast cancer

### Anthony Maxwell^1,2^, Nisha Sharma^3^, Hilary Dobson^4^, Sarah Vinnicombe^5^, Andrew Evans^5^

#### ^1^Nightingale Centre and Genesis Prevention Centre, University Hospital of South Manchester, Manchester, UK; ^2^Division of Informatics, Imaging and Data Sciences, School of Health Sciences, University of Manchester, Manchester, UK; ^3^Leeds Teaching Hospitals NHS Trust, Leeds, UK; ^4^West of Scotland Breast Screening Service, Glasgow, UK; ^5^University of Dundee, Ninewells Hospital & Medical School, Dundee, UK

##### **Correspondence:** Anthony Maxwell

**Introduction**

With increasing breast cancer incidence and improved survival, optimised follow-up strategies are required to maximise quality and duration of life. Whilst periodic mammography is adequate for most older women, its sensitivity in younger women and those with dense breasts is poor. Recurrences or new primaries in women whose original cancers were mammographically occult may also be occult. This study investigates the use of supplemental follow-up imaging in these groups.

**Methods**

An on-line survey of members of the British Society of Breast Radiology was performed, enquiring about the use of supplemental imaging follow-up, indications, modalities and numbers of women. One response only per hospital was requested. Categorical responses were reclassified where appropriate in line with submitted comments.

**Results**

Thirty-four valid responses from UK centres were received. Half treat >400 women with new breast cancers per year. Screen detected cancers comprise <25% of cases in 15% of centres and 25-75% in 85%. Additional imaging (mostly MRI) is offered by protocol/*ad hoc* in 0 and 8 (24%) units respectively for those with dense breasts, in 2 (6%) and 10 (29%) units for young women, and in 5 (15%) and 17 (50%) units for women with occult original cancers. Most perform <20 supplemental examinations per year. Local professional consensus and patient requests were the main reasons for providing additional imaging.

**Conclusion**

Practice varies widely between units. Mammographically occult original cancer is the commonest reason for supplemental follow-up imaging. Further research to ascertain its cost-effectiveness is required to develop evidence-based guidelines.

## PB.53 Male Breast Imaging – a local audit of referral practices and imaging

### David Grant, Briony Bishop, Roxanne Giggens, Shaheel Bhuva

#### Oxford University Hospitals NHS Foundation Trust, Oxford, UK

##### **Correspondence:** Shaheel Bhuva

**Introduction**

Our aim was to audit the referral of male patients to our unit, where all males, are imaged. For the purposes of this study, we used the following criteria to justify imaging:

i) Unilateral gynaecomastia (P2), >40 years
ii) P3-P5 or equivalent clinical description, any age

**Methods**

Retrospective review of all referrals for male breast imaging, over a 30-month period, from 01.01.14-30.06.16.

We recorded the referral source, clinical details including patient age, presenting symptoms and P value, imaging findings, and histological outcome.

**Results**

Over the 30-months, 518 males imaged. An increase in referral over this period amounting to >50% per annum.

Referral source: 322/518(62%) from breast surgery, 181/518(35%) GP and 15/518(3%) from other specialties.

Age: 13 to 93 years, bimodal peaks: 22-30 and 67-75years.

480/518 (92%) patients with P1/P2/clinically benign and U1/U2 and no biopsy. 4 patients, all >40 years, were P2U3B2.

20/518 (4%) patients biopsied – due to either clinical or imaging concerns.

1xB3 (in a P3U4 patient; no upgrade on sequent surgical excision) and 14 benign conditions (all U2/U3).

Remaining 18/518 patients, all P3U1/U2, were clinically reviewed +/- clinical core. All clinical cores were benign.

5/518 (1%) patients, all >70 years, all with P4/P5/clinically suspicious andU3-U5. Biopsy yielded malignancy: 4xB5b and 1xmelanoma.

30% justification rate according to audit standard criteria.

**Conclusion**

Multisource referral, increasing numbers and a cancer detection rate <1% in a department where all males are imaged. All clinically benign cases proved benign. All cancers were suspected. Findings strongly support the formulation of local guidelines.

## PB.54 Breast lesions incidentally detected by CT: Review of our local experience

### Jennifer Royds, Francesca Camilleri, Gauripriya Babu, Carolyn Beveridge

#### Mammography XRay, Edinburgh Breast Unit, Western General Hospital, Edinburgh, UK

##### **Correspondence:** Jennifer Royds

**Introduction/Methods**

Increasing use of body CT has lead to increasing detection of incidental breast lesions, creating two primary concerns within our unit:

1. that these patients experience longer delays before breast clinic review than standard symptomatic patients.
2. That some cases could avoid clinic altogether following specialist review of the CT/prior imaging.

49 cases over a 20 month period were reviewed to evaluate these concerns.

**Results**

Time between the triggering CT and clinic attendance varied from 1 to 163 days, inpatient range 1 - 19 days, median 9; outpatients range 6 - 163 days, median 24.

Of the 49 lesions, 36 were focal masses, 5 asymmetries, 5 enlarged nodes, 2 thickened WLE scars and one calcification. 31 biopsies were performed. 21 lesions (68%) were suspected and proven malignant (17 masses, 3 asymmetries, 1 scar recurrence). All lesions with ancillary suspicious findings were malignant. Hounsfield unit measurements were higher for malignant lesions.

5 patients had unchanged screening films or prior images. Breast radiologist review concluded 8 cases could have avoided clinic review as the area of concern was normal or classically benign.

**Conclusions**

1. Outpatients with incidental breast lesions experience greater delays before clinic review than the target 14 days.
2. Specialist current/prior imaging review could reduce unnecessary clinic assessment.

A high percentage (68%) of incidental lesions were malignant. We propose to develop a radiology:breast radiology referral pathway to address both these issues and improve standards of care for these patients at increased risk.

## PB.55 Are we over imaging the male breast?

### Rebecca Geach, Katherine Klimczak, Helen Massey, Iain Lyburn

#### Thirlestaine Breast Unit, Cheltenham, UK

##### **Correspondence:** Rebecca Geach

**Introduction**

Male breast cancer is rare affecting < 1% of the male population. We have observed a marked growth in the number of men referred for imaging and propose that imaging of all men may not be necessary.

**Method**

Retrospective review of all men referred for imaging between 2010 - 2015. Clinical history, imaging and pathology were reviewed.

**Results**

Total of 452 patients. 3 patients were referred for imaging in 2010 compared with 162 in 2015. In total, 11 patients were diagnosed with breast cancer, 2 with metastases from another primary and 3 with lymphoma.

Of these 16 patients, 2 were not given a numerical P value but the clinical history stated suspicious mass. 12 (75%) had a P value of P3-P5. 2 patients were clinically P2 but both had significant history: 1 of blood stained nipple discharge and the other of previous renal cell carcinoma.

The average age of the 16 patients was 75 years, the youngest 53 years.

In 2015, 84 (52%) patients referred for imaging were clinically graded P2 gynaecomastia confirmed on ultrasound alone (U2). 10 patients were clinically P2 gynaecomastia, ultrasound was indeterminate (U3) but core biopsy confirmed gynaecomastia. The incidence of malignancy was 3%.

**Conclusion**

454 men were referred for imaging over 5 years. Malignancy was detected in 3.5%. Of those patients with malignancy, 88% were clinically suspicious and all were >50 years. We propose an imaging pathway for men based upon clinical findings/age that would be safe and reduce unnecessary imaging and biopsy.

## PB.56 Are Asian women more prone to less favourable subtypes of breast cancer?

### Joseph Wan^1^, Anil Jain^2,3^, Jaanilka Molderson^2^

#### ^1^University of Manchester, Manchester, UK; ^2^The Nightingale Centre and Genesis Prevention Centre, University Hospital of South Manchester, Manchester, UK; ^3^Manchester Academic Health Sciences Centre, Institute of Cancer Sciences, Manchester, UK

##### **Correspondence:** Joseph Wan

**Introduction**

Breast cancers present with differential expression levels of hormone receptors (ER, PR, HER2), which modern chemotherapy targets, resulting in 8 different subtypes. Research has shown that each subtype presents with different histopathological features, thus affecting survival. We aim to investigate the prevalence of breast cancer receptor subtypes between Asian and Caucasian women; and to compare their prognostic indices.

**Methods**

231 Asian patients with invasive breast carcinoma (IBC), age-matched with equal numbers of Caucasian patients; are included in this retrospective cohort study (n=462). Receptor profiles and histopathological features for each case have been extracted from medical records along with calculation of Nottingham Prognostic Index (NPI).

**Results**

Across all receptor subtypes, Asian patients presented with higher NPI; lymph node status; invasive grade; and larger tumour sizes (p<0.005). They have higher prevalence of HER2-positive subtypes (30.3%, p=0.002); whereas Caucasian patients have a higher proportion of ER+/PR+/HER2- subtype (p=0.030), which is associated with favourable prognostic indices. TNBC prevalence is similar in both groups, demonstrating the highest proportion of grade 3 tumours, while HER2-positive subtypes demonstrated the highest NPI. Furthermore, prognostic indices were worse in younger patients.

**Conclusion**

Consistent with recent literature, our study provides evidence that Asian patients are more prone to HER2-overexpressing breast cancer, but not TNBC subtypes, Also, poor clinico-pathological features were associated with these 2 subtypes, correlating to a poor prognosis. Given the poor prognostic indices they present with and high treatment costs involved with these subtypes, our results have implications for clinical care and future research.

## PB.58 Extended Coverage CT vs Bone Scan for staging of bony metastases in locally advanced or aggressive breast cancer - Cost efficiency and savings in current NHS practice

### Seema Salehi-Bird, Sahithi Nishtala, Liz Gunning

#### University Hospital North Midlands, North Midlands, UK

##### **Correspondence:** Seema Salehi-Bird

**Background**

Our institution performs Tecnitium-99m bone scan in addition to CT thorax, abdomen and pelvis as a part of routine staging of patients with locally advanced or aggressive breast cancer. The purpose of this study is to 1) determine if bone scan offers any additional information over CT and 2) evaluate the potential cost effectiveness of performing extended coverage CT alone, without additional bone scan.

**Method**

Retrospective review of cases at a single university hospital for a period of 30 months was undertaken. Inclusion criteria: CT and bone scan within 2 weeks of each other with a new diagnosis of breast cancer. Exclusion criteria: previous malignancy, symptomatic patients, contralateral breast malignancy.

**Results**

113 patients included, all female.

In the bone scan group there were 5 false negatives and 1 false positive. In the CT group there was 1 false negative.

**Conclusion**

Our experience shows that CT is highly accurate in staging asymptomatic women with suspected bony metastasis. Staging CT picked up 95% of bony metastatic lesions. Bone scan failed to identify 5 (23%) bone metastases and overcalled a single diagnosis of metastasis. At our trust a Tc-99m bone scan costs £246 and is significantly more expensive than CT costing £147. There is huge potential cost saving by removing bone scan from routine work up of this patient group. Instead, extended coverage CT (base of skull to mid femur) at a cost of £164 may be performed. This will reduce overall radiation dose to the patient and is economically beneficial.

## PB.59 Is routine use of Bone scan appropriate in asymptomatic breast cancer patients with suspected metastasis?

### Deepthi Vinayan Changaradil, Jyoti Bansal

#### University Hospital Of Llandough, Llandough, UK

##### **Correspondence:** Deepthi Vinayan Changaradil

**Background**

Royal College of Radiologists guidelines from 2013 recommends contrast enhanced CT of chest, abdomen and pelvis for breast cancer patients with suspected metastasis, which may obviate the need for bone scan in asymptomatic patients.

**Aim**

The purpose of this study is to perform a head to head comparison of bone scan and CT scan in breast cancer patients. It aims to evaluate if any extra bony lesions were picked up with bone scan, and assess the number of false negative bone scan or CT scans, if any.

Method: Between June 2006 and January 2016, 164 breast cancer patients had staging investigations (either both CT and bone scans or bone scan only or CT only). All images and reports on PACS were evaluated retrospectively.

**Results**

104/164 patients had both CT and bone scan within one month of each other. Among these 104, 48 had both investigations completely normal while 14 had concordant abnormal findings.

CT picked up extra metastatic soft tissue findings(mostly lung or liver) in 25(23.8%). Bone scans picked extra abnormal findings in 5(4.8%),either skull or peripheral bony metastasis, but all 5 had other metastatic lesions visible on CT, either within axial skeleton or soft tissues. CT and bone scan had equivocal findings in 14(13.4%) and 12(11.5%) patients respectively. There were 3(2.8%) false negative bone scans.

**Conclusion**

CT picked up substantial number of extra soft tissue metastasis. Bone scan did not add to the total number of metastatic patients. Our findings suggest routine bone scan in asymptomatic patients with breast cancer is inappropriate.

## PB.60 Audit of double reading and arbitration of long term follow up recalls

### Joanna Davis, Ashwini Sharma, Elena Del Voscovo

#### Western General Hospital, Edinburgh, UK

##### **Correspondence:** Joanna Davis

**Background**

Longterm follow up mammograms of patients post surgery form a considerable bulk of the reporting in a symptomatic breast unit. Recalls from this group add considerably to patient anxiety, and contribute to workload, taking up valuable appointment time for spot views, ultrasound and biopsy.

**Aim**

A decision was made to second read all recalls from the LTFU group; arbitration by a third consultant was used as a deciding vote in case of disagreement. All second reads and arbitrations were recorded in a diary - this has been ongoing for a year. All cases were analysed for presence or absence of malignancy when recalled, retrospectively.

**Results**

Of 93 LTFU patients recalled from June 2015 to May 2016, 77 patients were recalled after arbitration. 58 of these patients (75.3%) were returned to routine followup following arbitration; 19 patients (24.7%) were recalled following arbitration. 3 malignant cases (16%) were noted in the recall group post arbitration; 16 cases (84%) were benign. The most commonly arbitrated lesion was opacity, in 45%; 37% were recalled for an opacity.

**Conclusion**

Second reading with arbitration, as in screening, is a valuable tool to reduce number of recalls in the LTFU group, allowing 75% of arbitrated cases to be returned to normal followup. This reduces patient anxiety, and frees up valuable time for assessment of true positive malignant recalls. In the absence of established guidelines, the screening recall rates of 4% for incident round and 7% for prevalent round can be used to compare symptomatic recall rate in an instutution.

## PB.61 Impact of Digital Breast Tomosynthesis (DBT) as a standard mammographic investigation compared with Full Field Digital Mammography (FFDM) in a District General Hospital Symptomatic Breast Service

### Harriet Etheridge^3,2^, Aisling Butler^1,2^

#### ^1^Neath Port Talbot Hospital, Baglan, UK; ^2^The Princess of Wales Hospital, Bridgend, UK; ^3^University of Birmingham Medical School, Birmingham, UK

##### **Correspondence:** Aisling Butler

**Introduction**

Digital Breast Tomosynthesis is established as a problem-solving tool within NHS screening. Recent publications have examined the impact of DBT as an assessment tool within the symptomatic setting. To date, literature has not reported on the impact of DBT as a baseline study in this scenario although internationally many centres have adopted this practice. Our centre installed a Hologic Dimensions Tomographic unit in May 2015. Upon installation, DBT replaced Full Field Digital Mammography (FFDM) as the baseline tool for symptomatic and surveillance patients.

**Aim**

To establish if the number of M3 reports have increased or decreased and to quantify the positive predictive value of M3 reports.

**Methods**

DBT mammographic reports over 12months were reviewed and compared with FFDM reports in the previous 12month period, evaluating ‘M3' reports and their subsequent up- or downgrading. Cases were evaluated to identify the following - additional mammographic views, ultrasound grading, histopathological classification and final diagnosis.

**Results**

There were less M3 reports overall. Of the M3 reports, more were true positive than in the previous year. Within this specific cohort we identified a 10.10% increased likelihood of ultrasound upgrade following DBT compared with FFDM, a 25.7% increased likelihood of histological upgrade and a 7.1% increase in ability to predict malignancy using DBT compared to FFDM.

**Discussion**

We acknowledge the limitations in terms of design and cohort but the results of this small centre study are in line with previous screening studies and substantiate the use of DBT as a baseline tool in the symptomatic setting.

## PB.62 Imaging and male breast cancer: Should mammography be employed first-line? How common is breast cancer in men imaged for gynaecomastia?

### David S J Fenne^1^, Gary Rubin^1,2^

#### ^1^Brighton and Sussex Medical School, Brighton, UK; ^2^The Park Centre for Breast Care, Brighton, UK

##### **Correspondence:** David S J Fenne

**Introduction**

Traditionally mammography has been used as the first-line imaging test in patients with breast symptoms aged over 40 years of either sex. Some centres advocate ‘ultrasound first’ in men instead. This study assessed whether mammography correctly identified cancers in a ‘mammography first’ unit, the imaging modalities used and the relationship of gynaecomastia to imaging and breast cancer.

**Methods**

We looked at male breast cancers from 2010-2014, in addition to the number of men referred to our breast unit who were imaged with mammography, ultrasound, or both in 2014, noting whether they had gynaecomastia.

**Results**

From 2010-2014, 19 men had breast cancer, 2 bilaterally. All were detected on imaging, including 2 that were unsuspected clinically. Only 1 occurred in a man with clinically simple gynaecomastia. In 2014, 207 men aged 40+ came to our unit and 143 were imaged. 71 had mammography without ultrasound, 63 had both and 9 just ultrasound. Gynaecomastia was the most common indication and finding, being seen in 95 men, representing 68% of those imaged. Assuming 2014 was representative of 2010-2014, 475 men with gynaecomastia needed imaging to find one unsuspected breast cancer, a lower detection rate than in the NHS breast screening programme.

**Conclusions**

Neither mammography nor ultrasound missed or misclassified any cancers. This study supports using mammography or ultrasound first-line to image men aged 40+, depending on local resources. Gynaecomastia was the major imaging indicator/finding. There should be stricter adherence to guidelines that limit breast imaging for gynaecomastia, as it rarely masks cancer.

## PB.63 Extra Extra read all about it. Additional requests for imaging in the Breast Unit

### Helen Burt, Nick Ridley, Sarah Taylor

#### Breast Unit Great Western Hospital, Swindon, UK

##### **Correspondence:** Nick Ridley

**Introduction**

The primary imaging workload in the breast unit is within the context of the symptomatic fast track clinic or screening assessment clinic. These usually have defined numbers of patients. In addition there is a significant increasing workload of extra patients who require imaging and invasive procedures via other pathways, which we aimed to quantify.

**Methods**

The data was identified retrospectively from the Computerised Radiology Information System (CRIS) and recorded on a spreadsheet.

Information was collected on the number of extra patients who were imaged and reviewed by the Breast Radiologists in addition to their booked workload during the months of May and June 2016.

**Results**

Over a two month period there were an additional 60 patients. These patients were referred from a wide variety of departments within the hospital, although the majority were referred through the breast surgeons.

A total of 48 ultrasound examinations and 21 mammograms were performed and reported on these patients. The additional procedures performed were also recorded, comprising 6 core biopsies, 2 fine needle aspirations, 4 seroma aspirations and 3 abscess drainages.

**Conclusion**

There is a significant “extra” workload performed during the working week by Breast Radiologists which may not be reflected in funding streams or be accounted for in job plans. Extra imaging represents a significant workload; the equivalent of 3-4 symptomatic clinics a month. With escalating trends for imaging and a greater reliance on image-guided seroma and abscess drainage, it is likely that this will increase.

## PB.64 Male breast patient imaging: what is an appropriate pathway?

### Muthyala Sreenivas

#### UHCW NHS Trust, Coventry, UK

**Background**

Breast cancer in much less common in men when compared to women and many cancers occurs in men over 50 years of age. Routine usage of imaging in men presenting via fast track clinic may be not be appropriate and a better stratification of patients for imaging is warranted.

**Methods**

Retrospective data between 2010 to 2014 from hospital RIS identified a total of 500 male patients of varying ages who were all referred for imaging. Various patient demographic details were recorded and outcomes of triple assessment correlated.

**Results**

Average age of the patients was 45 years. There was no consistency as to what imaging was used. 130 patients received US only, 12 received mammograms only and 358 received both US and mammograms. 54 US core biopsies and one stereocore biopsy was performed. 488 patients were scored P2 on clinical examination of which none had a cancer and imaging and pathology findings (where necessary) were benign. 8 were scored P3 and there were two cancers in this group. All three P4 cases were benign on imaging and pathology. There was a single P5 case which was malignant on imaging and on pathology. Overall three malignancies were detected which were all in men over 70 years of age.

**Conclusion**

Routine imaging of men is not warranted. An alternate more cost effective pathway was devised and is being implemented at UHCW.

## PB.65 Impact of limiting symptomatic mammograms and use of ultrasound as first line investigation in younger women (35-40years) presenting with benign/indeterminate breast symptoms -- Evaluating the local practice

### Subodh Seth^1^, Archana Seth^1,2^

#### ^1^Forth Valley Royal Hospital, Larbert, Stirlingshire, UK; ^2^West of Scotland Breast Screening Services, Glasgow, UK

##### **Correspondence:** Archana Seth

**Introduction**

Since 2012, in keeping with ‘Best Practice Guidelines’ Forth Valley breast-team has changed the practice and we don’t use mammograms as regular adjunct to assessment in women in age-group 35-40years. Not all centres in Scotland are following this guideline and this results in anxiety over potential missed cancer diagnosis.

**Methods**

Caldicott approval was obtained. List of breast cancers diagnosed in year 2013 was obtained from the local audit department. Radiology database and clinical portal were searched for relevant breast imaging and clinical information.

**Results**

3610 women were imaged in year 2013 and 242 cancers were diagnosed.

310 of these women were in the age-group 35-40 years and 11 cancers were diagnosed.

Analysing the data for these 11 cases, 9 had abnormal clinical and ultrasound findings. Mammograms were not crucial to reach the diagnosis.

1 case was clinically benign but had suspicious ultrasound findings. Lesion was occult on subsequent mammograms.

In the remaining case, only abnormality was incidental lymphnode which was metastatic on biopsy. Malignant calcification was seen on subsequent mammography. This is the only case where potentially we could have missed the diagnosis.

**Conclusion**

In this study, almost all cancers were picked-up on clinical-examination or ultrasound. There was a single case where without upfront mammography we could potentially have missed the diagnosis of cancer. This was picked-up on diligent scanning of the whole quadrant.

In our practice, it is safe to limit the use of mammography in the age-group 35-40years and continue using ultrasound as the first line investigation.

